# Cytochrome P450-dependent biotransformation capacities in embryonic, juvenile and adult stages of zebrafish (*Danio rerio*)—a state-of-the-art review

**DOI:** 10.1007/s00204-021-03071-7

**Published:** 2021-06-20

**Authors:** Ann-Kathrin Loerracher, Thomas Braunbeck

**Affiliations:** Aquatic Ecology and Toxicology Section, Centre for Organismal Studies, University of Heidelberg, Im Neuenheimer Feld 504, 69120 Heidelberg, Germany

**Keywords:** Cytochrome P450, Biotransformation, Xenobiotic metabolism, Zebrafish, Embryo, Ecotoxicology, Toxicology

## Abstract

Given the strong trend to implement zebrafish (*Danio rerio*) embryos as translational model not only in ecotoxicological, but also toxicological testing strategies, there is an increasing need for a better understanding of their capacity for xenobiotic biotransformation. With respect to the extrapolation of toxicological data from zebrafish embryos to other life stages or even other organisms, qualitative and quantitative differences in biotransformation pathways, above all in cytochrome P450-dependent (CYP) phase I biotransformation, may lead to over- or underestimation of the hazard and risk certain xenobiotic compounds may pose to later developmental stages or other species. This review provides a comprehensive state-of-the-art overview of the scientific knowledge on the development of the CYP1-4 families and corresponding phase I biotransformation and bioactivation capacities in zebrafish. A total of 68 publications dealing with spatiotemporal CYP mRNA expression patterns, activities towards mammalian CYP-probe substrates, bioactivation and detoxification activities, as well as metabolite profiling were analyzed and included in this review. The main results allow for the following conclusions: (1) Extensive work has been done to document mRNA expression of CYP isoforms from earliest embryonic stages of zebrafish, but juvenile and adult zebrafish have been largely neglected so far. (2) There is insufficient understanding of how sex- and developmental stage-related differences in expression levels of certain CYP isoforms may impact biotransformation and bioactivation capacities in the respective sexes and in different developmental stages of zebrafish. (3) Albeit qualitatively often identical, many studies revealed quantitative differences in metabolic activities of zebrafish embryos and later developmental stages. However, the actual relevance of age-related differences on the outcome of toxicological studies still needs to be clarified. (4) With respect to current remaining gaps, there is still an urgent need for further studies systematically assessing metabolic profiles and capacities of CYP isoforms in zebrafish. Given the increasing importance of Adverse Outcome Pathway (AOP) concepts, an improved understanding of CYP capacities appears essential for the interpretation and outcome of (eco)toxicological studies.

## Cytochrome P450-dependent biotransformation in (eco)toxicology

“Xenobiotic biotransformation” refers to the process by which a compound foreign to an organism is converted into a usually more polar, i.e. more hydrophilic, and thus more readily excretable metabolite (Parkinson et al. 2013). Biotransformation is conventionally divided into two distinct phases: Phase I reactions are functionalization reactions, which serve to incorporate functional groups into the molecular structure of a xenobiotic compound or expose (demask) already existing polar groups (Parkinson and Ogilvie 2008; Penner et al. 2012). This is achieved via oxidation, reduction or hydrolysis reactions catalyzed, e.g., by alcohol dehydrogenases, epoxide hydrolases, flavin-containing monoaminooxidases and by cytochrome P450-dependent monooxygenases (CYPs; Chen 2020; Penner et al. 2012; Testa 2008). Phase I metabolites may be the final products ready for excretion, but usually undergo phase II biotransformation in which already existing, incorporated or exposed functional groups serve as active sites for conjugation with endogenous polar molecules (Parkinson et al. 2013).

CYP enzymes constitute a functionally diverse superfamily of cysteine thiolate-ligated heme enzymes. Present in most tissues and organs, CYPs are by far the predominant phase I biotransformation enzymes. They catalyze aromatic and aliphatic hydroxylation, azo reduction, desulfuration, epoxidation, *N*-hydroxylation, *O*- and *N*-dealkylation, nitro reduction, oxidative dehalogenation and sulfoxidation on a broad range of compounds, ranging from small non-polar molecules to complex polypeptides (Guengerich 2001; Isin and Guengerich 2007; Qiang and Lu 2014).

Based on amino sequence similarities, CYPs are clustered into CYP families and subfamilies. CYPs sharing a minimum of 40% amino sequence similarity are grouped within the same CYP family (e.g., CYP1, CYP2, CYP3), and those sharing at least 55% similarity are grouped within a CYP subfamily (e.g., CYP1A, CYP2B, CYP3C; Nelson 2006; Nelson et al. 1993). Whereas most isoforms of the CYP families 1, 2, 3 and 4 primarily act on xenobiotic compounds (Nebert and Russell 2002; Rendic and Guengerich 2015; Taavitsainen 2002), most isoforms of the CYP families 5–51 predominantly act on endogenous substrates, many of which have critical roles in normal development, maturation and physiological homeostasis (Guengerich 2017; Nebert et al. 2013).

CYPs do not only modify physicochemical characteristics of their substrates, but may also impact their (eco)toxicological properties. Given the broad spectrum of substrates accepted by at least part of the CYP isoforms, the competition of different CYP isoforms for substrates and the complexity of CYP-mediated transformations, CYPs are involved in both detoxification and toxification processes: CYPs usually increase the polarity of xenobiotic compounds and enable phase II biotransformation and, thereby, contribute to an efficient excretion and detoxification (Guengerich 2003; Suter 2008). However, there are numerous examples for CYP-catalyzed reactions which result in the formation of metabolites or intermediates that are more reactive, teratogenic or even carcinogenic and/or toxic than the parent compounds (i.e. pro-carcinogens, protoxicants and proteratogens), a process referred to as xenobiotic bioactivation (Smith and Brian 1991; Stiborova et al. 1992; Weigt et al. 2011).

Over the past two decades, the zebrafish *(*zf, *Danio rerio*) and − specifically − zebrafish embryos (≤ 120 h post-fertilization; hpf) have emerged as pre-eminent model organisms with numerous applications not only in ecotoxicology, but also in toxicology and pharmacology (Bambino and Chu 2017; Barros et al. 2008; Hill et al. 2005; Kithcart and MacRae 2017; McGrath and Li 2008). At least in Europe, early developmental stages of zebrafish have received particular attention, since these are not regarded protected according to current EU animal welfare legislation (EU 2010; Strähle et al. 2012). Current examples of the use of zebrafish embryos within validated test protocols include the fish egg test (DIN 38,415–6; ISO 2016), which is a mandatory stand-alone component in routine whole effluent toxicity testing in Germany (Bundesgesetzblatt 2005; Norberg-King et al. 2018), and the fish embryo toxicity test (FET, OECD TG 236), which was originally designed for determination of the acute toxicity of chemicals on embryonic stages of fish (Busquet et al. 2013), be it as a full replacement for the acute fish toxicity test (AFT; OECD; TG 203) conducted with juvenile and adult (zebra)fish to provide data for regulatory decision making (Braunbeck et al. 2015; Scholz et al. 2013) or as a central component of weight-of-evidence approaches (ECHA 2017; Lillicrap et al. 2020; Moe et al. 2020; Paparella et al. 2021). In fact, the identification of a number toxicological outliers (i.e. ~ 30 compounds with more than ten times lower toxicity in the FET than in the AFT, e.g., allyl alcohol, cyclohexane, nonylphenol, dieldrin and permethrin (Klüver et al. 2014, 2015) has drawn attention to potential limitations of the FET and has led to criticism concerning its regulatory use as surrogate for the AFT (Sobanska et al. 2018).

Concerns have been raised that, due to potential limitations in phase I biotransformation and bioactivation capacities of zebrafish embryos, there might be a risk of underestimation the toxicity that pro-toxicants might pose to juvenile or adult fish, but not to embryos (Busquet et al. 2008; Saad et al. 2016b; Verbueken et al. 2017). Since then, there is an ongoing debate whether or not zebrafish embryos have sufficient capacities to biotransform and bioactivate xenobiotics. The clarification of this aspect is, however, of particular relevance for our understanding of the capabilities and limitations that eventually define the applicability domain of the FET (Sobanska et al. 2018).

Over the past two decades, the CYP system, and in particular the 56 isoforms identified for the zebrafish CYP families 1, 2, 3 and 4 (Table 1), have been subject to many studies that have led to a more detailed and sophisticated understanding of the development of the CYP-dependent biotransformation and bioactivation capacities in zebrafish. These studies provided insights into developmental CYP mRNA expression patterns, tissue- and organ distribution of CYP transcripts, metabolic activities towards fluorogenic and luminogenic mammalian CYP probe substrates, bioactivation activities towards pro-toxicants and pro-teratogens and xenobiotic metabolite formation. This review provides an in-depth coverage of the current state-of-knowledge on the CYP1-4 families and phase I biotransformation and bioactivation capacities of zebrafish in general, and zebrafish embryos in specific. Data will be critically discussed and, whenever possible, compared with findings for other fish species. Thereby, this review will identify gaps in our knowledge and discuss future directions for research.

**Table 1:** List of zebrafish CYP1, CYP2, CYP3 and CYP4 genes (GRCz11 assessed by Ensembl genome browser; release 100)

CYP1	CYP2				CYP3	CYP4
CYP1A	CYP2AA1	CYP2AD3	CYP2K20	CYP2V1	CYP3A65	CYP4F3
CYP1B1	CYP2AA2	CYP2AD6	CYP2K21	CYP2Y3	CYP3C1	CYP4T8
CYP1C1	CYP2AA3	CYP2AE1	CYP2K22	CYP2X6	CYP3C2	CYP4V7
CYP1C2	CYP2AA4	CYP2J20	CYP2N13	CYP2X7	CYP3C3	CYP4V8
CYP1D1	CYP2AA6	CYP2K6^*^	CYP2P6	CYP2X8	CYP3C4	
	CYP2AA7	CYP2K6^**^	CYP2P7	CYP2X9		
	CYP2AA8	CYP2K8	CYP2P8	CYP2X10.2^+^		
	CYP2AA9	CYP2K16	CYP2P9	CYP2X10.2^++^		
	CYP2AA11	CYP2K17	CYP2P10	CYP2X12		
	CYP2AA12	CYP2K18	CYP2R1			
	CYP2AD2	CYP2K19	CYP2U1			

*ENSDARG00000098995, ^**^ENSDARG0000009874, ^+^ENSDARG60000006501, ^++^ENDSARG600000068283

## Literature sources used for this review

Relevant studies were identified through searching the following databases: Science Direct, PubMed, Scopus, Google Scholar and Web of Science using the keywords bioactivation, biotransformation, cytochrome P450, CYP, *Danio rerio*, expression, fish, metabolism, metabolite, phase I, pro-teratogen, pro-toxicant, pro-mutagen, xenobiotic and zebrafish as well as combinations thereof. In addition, reference lists of relevant articles were manually searched for further potentially relevant publications. No restrictions were made regarding the date and language of the publication. The searches were undertaken between November 2017 and March 2021 and yielded an array of publications on the cytochrome P450 system and phase I biotransformation, including peer-reviewed studies, reviews, original research articles and academic theses.

## Spatial and temporal CYP gene expression patterns

**Fig. 1:**
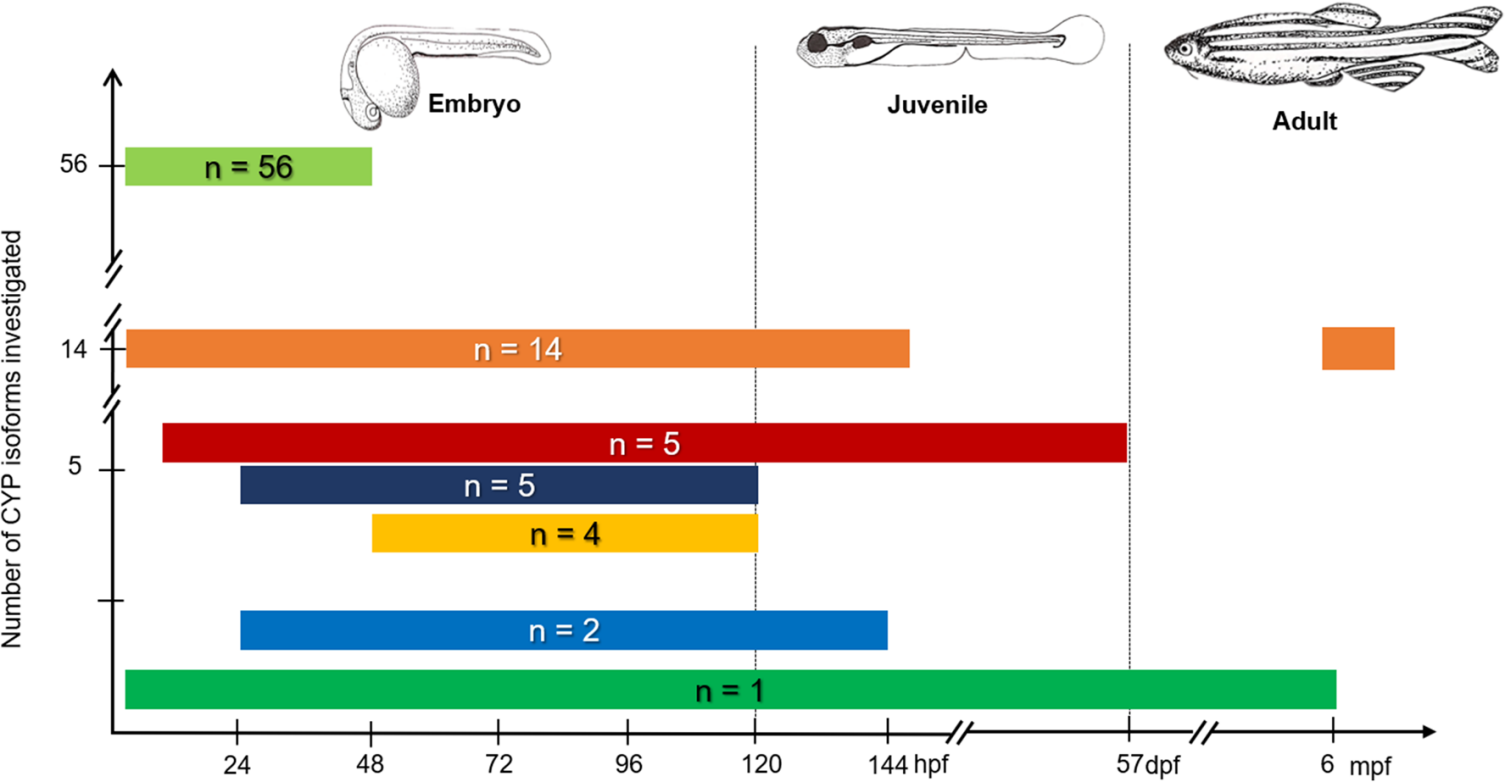
Numbers of CYP1-4 isoforms whose constitutive expression patterns have been determined throughout embryonic, juvenile and adult development of the zebrafish (*Danio rerio*). Illustrations by Karlotta Boßung

Most of the current knowledge regarding CYP-dependent phase I biotransformation capacities in different developmental stages of zebrafish has been derived from studies profiling temporal (i.e. developmental) and spatial (i.e. tissue and organ distribution) mRNA expression patterns of the 56 genes identified for the zebrafish CYP families 1, 2, 3 and 4 (Saad et al. 2016a). These studies have built up a comprehensive data set, comprising information on developmental expression patterns of the full complement of zebrafish CYP1-4 genes as well as on organ- and tissue-specific expression patterns of 20 CYP1-4 genes. Figure 1 gives an overview of the number of CYPs whose temporal trends in constitutive expression levels have been studied in zebrafish across different stages of development (for details, Table 2). Table 3 presents a collection of organ- and tissue-specific CYP expression patterns reported for embryonic (≤ 120 hpf), juvenile (≥ 120 hpf, ≤ 3 mpf) and adult zebrafish (> 3 mpf), respectively.

**Table ﻿2:**
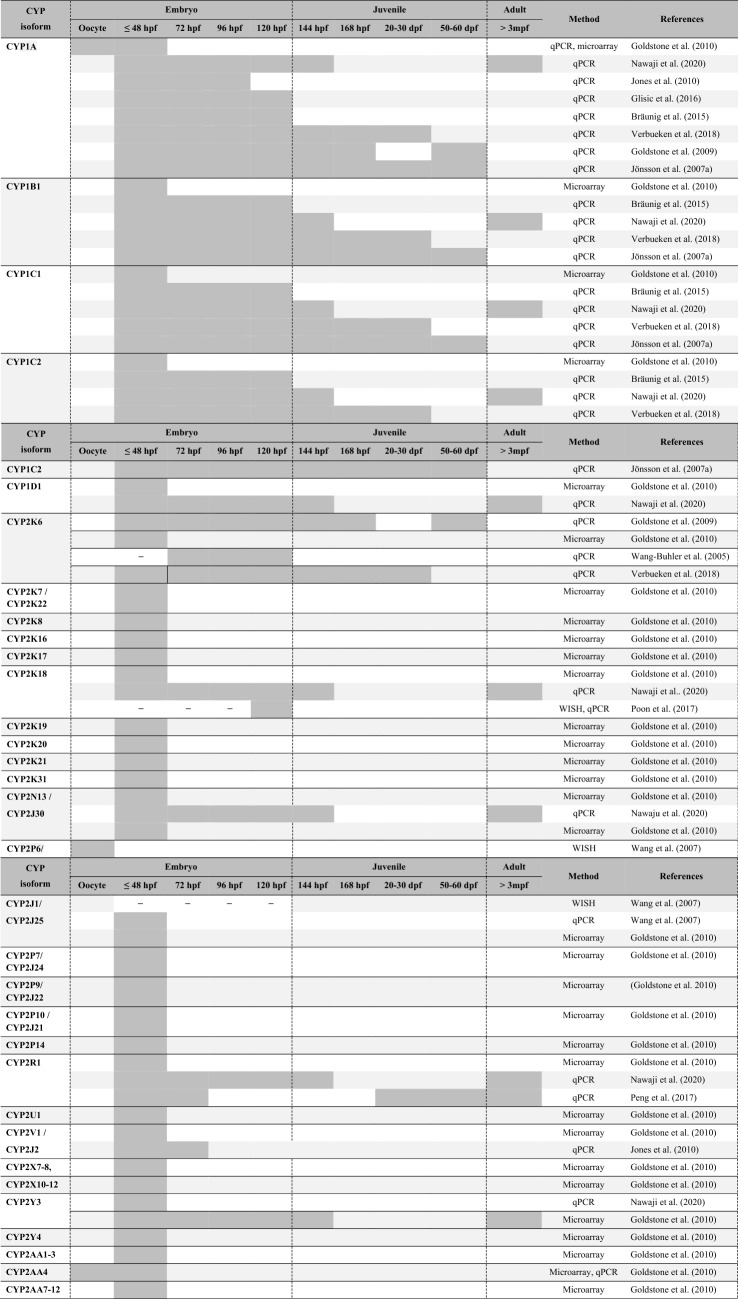

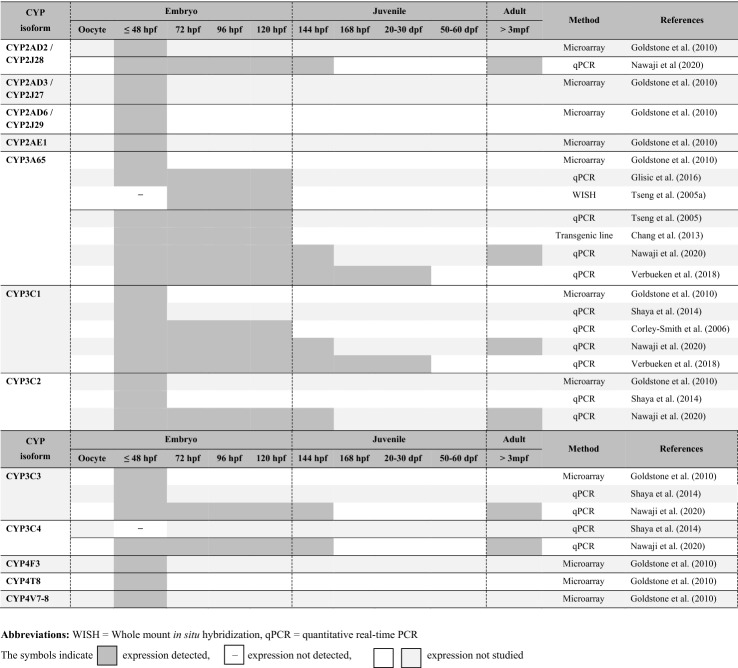
Information available on the mRNA expression of CYP1, CYP2, CYP3 and CYP4 genes in embryonic, juvenile and adult zebrafish (*Danio rerio*)

**Table 3:** Tissue and organ distribution of zebrafish CYP1, CYP2, CYP3 and CYP4 families. mRNA transcripts in embryonic, juvenile and adult stages of zebrafish (*Danio rerio*)

CYP	Age	Head			Trunk							Method	Reference
		Brain	Eye	Gill	Heart	Intestine	Kidney	Liver	Gonad		Other		
CYP1A	Embryo (30 hpf)	+	+ + +		+ + +	+ + +				+ + +	Skin, pectoral fin bud, cloaca, intersegmental vessels	ISH	Kim et al. (2013)
										+	Otic vesicle		
	Juvenile (21 dpf)	−	−		+	+ + +	+ +			+ + +	Nose, oropharynx, pseudobranch, skin (head)	IHC	Taylor (2005)
										+ +	Esophagus, taste bud		
	Adult	( +)	( +)	+	+	+ +	+ +	+ + +				qPCR	Goldstone et al. (2009)
	Adult	( +)	+	+	+	+ +	+	+ + +	( +)			qPCR	Jönsson et al. (2007a)
CYP1B1	Embryo (24–96 hpf)	+	+				−					WISH	Yin et al. (2008)
	Adult	+ + +	+ + +	+ +	+ + +	+	+ +	+ +	+			qPCR	Jönsson et al. (2007b)
CYP1C1	Adult	+ +	+ +	+ +	+ + +	+	+	+	♂ + ♀ ( +)			qPCR	Jönsson et al. (2007b)
CYP1C2	Adult	+ +	+ +	+	+ +	+	+ +	+	♂ + ♀ ( +)			qPCR	Jönsson et al. (2007b)
CYP1D1	Adult	+	( +)	+	+	+	+	+ + +				qPCR	Goldstone et al. (2009)
CYP2J1	Adult	+ +			+ + +	–	+ + +	+ +	♂ + ♀ + +			qPCR	Wang et al. (2007)
CYP2K6	Juvenile (21 dpf)	−	+ +	−	+ / + +	–	+ + +	-		+	Skin, oropharynx, esophagus	IHC	Taylor (2005)
										+ + +	Muscle tunic of the intestine		
	Adult	−	−	−	−	−		** + + + **	**♂ -** **♀ + + + **			qPCR	Wang-Buhler et al. (2005)
CYP2K7/CYP2K22	Embryo (96 hpf)	−	−	−	−	−	+	–				WISH	Fetter et al. (2015)
	Juvenile (21 dpf)		+	+ + +		+ + +	+			+ +	Skin (head), oropharynx, esophagus	IHC	Taylor (2005)
										+ + +	Taste bud, skin (trunk), cartilage		
CYP2K18	Embryo (120 hpf)					+						TL	Poon et al. (2017a)
		−	−	−	−	+	−	+				WISH	
CYP2N13	Embryo (120 hpf)	−	−	−	−	+	−	+				WISH	Poon et al. (2017a)
						+						TL	
					+			+		+	Olfactory bulb, cloaca, skin	TL	
CYP2R1	Adult	−	−	−	−	−	−	+ + +	♂ − ♀ + +	+ + +	Adipose tissue Muscle	qPCR	Peng et al. (2017)
CYP2Y3	Embryo (55 hpf)					+		+				WISH	Nawaji et al. (2020)
CYP2AA1	Adult ♀	+	+		+	+ + +	+	+	+			qPCR	Kubota et al. (2013)
	Adult ♂	+	+		+ +	+ + +	+ +	+ +	+ +				
CYP2AA2	Adult ♀	+	+		( +)	+	+ + +	+ +	( +)			qPCR	Kubota et al. (2013)
	Adult ♂	+	+		( +)	+ +	+ + +	+	( +)				
CYP3A65	Embryo (72 hpf)	–	−		−	−	−	+				WISH	Tseng et al. (2005b)
	Embryo (84 hpf)	–	−		−	+	−	+ +					
	Embryo (96 hpf)	–	−		−	+ +	−	+ +					
	Embryo (120 hpf)	–	−		−	+ + +	−	+ + +					
	Juvenile (21 dpf)	–	+ +	-	+	+	+	−		+ + + + + +	Skin (trunk), skeletal muscle Ear Corpuscle of Stannius	IHC	Taylor (2005)
	Adult	( +)	( +)	+	+	+ + +		+ + +				qPCR	(Tseng et al., 2005)
CYP3C1	Embryo (12 hpf)		Widely distributed through the whole body									WISH	Corley-Smith et al. (2006)
	Embryo (48 hpf)	+ + +		Widely distributed									
	Embryo (120 hpf)	+ + +				+ + +				+ + +	Pharynx		
CYP3C1	Juvenile (21 dpf) Juvenile (21 dpf)		+ + +	+ + +		+ +	+ + +	+		+ + + + + +	Skin, ear, taste bud, pharyngeal mill Neurons, skin Pseudobranch, oropharynx	IHC	Taylor 2005)
	Adult	–	+	+	+	+ + +		+ + +	♀ + + +	+	Skin	qPCR	Corley-Smith et al. (2006)
	Adult ♀	+ +	+	+	+ +	+ +	( +)	+	+ + +	+	Olfactory rosette, spleen	qPCR	Shaya et al. (2014)
	Adult ♂	+	+ + +	+	+	( +)	+ +	+ + +	+ +	+ + + +	Spleen Olfactory rosette		
CYP3C2	Adult ♀	+	+	+	+	+ + +	( +)	+	+ +	+	Spleen, olfactory rosette	qPCR	Shaya et al. (2014)
	Adult ♂	+	+	+	+	+	+	+	+ +	+	Spleen, olfactory rosette	qPCR	
CYP3C3	Adult ♀	+	( +)	( +)	+	+	+	+	+	( +) +	Spleen Olfactory rosette	qPCR	Shaya et al. (2014)
	Adult ♂	( +)	( +)	( +)	+	+ + +	( +)	+	+	( +)	Olfactory rosette, spleen	qPCR	
CYP3C4	Adult ♀	+	+	+ +	+	+	+ +	+	+ +	+	Olfactory rosette, spleen	qPCR	Shaya et al. (2014)
	Adult ♂	+ + +	+ + +	+	+	+ + +	+	+	+	+	Olfactory rosette, spleen	qPCR	

Expression levels of CYP genes: “ +  +  + ” high, “ +  + ” moderate, “ + ” minor, “( +)” negligible,—not detected, “no entry” not studied

*IHC* immunohistochemistry, *ISH* in situ hybridization, *qPCR* quantitative real-time PCR, *TL* transgenic line, *WISH* whole mount in situ hybridization

Besides the use as an indicator of metabolic competence, data on spatiotemporal CYP 1–4 expression patterns can also be informative on characterizing functional (i.e. physiological) roles of CYP isoforms in zebrafish. For instance, the relatively high CYP3C1 expression level found in the brains of 48 and 120 h old zebrafish embryos (Corley-Smith et al. 2006), the lack of CYP3C1 mRNA in the brains of 21 d old zebrafish (Taylor 2005), and its prominent expression in the main xenobiotic-metabolizing organs, i.e. intestine and liver, in adult zebrafish (Corley-Smith et al. 2006) make it is reasonable to assume that the function of the CYP3C1 might not only be related to xenobiotic biotransformation, but also to brain development in early life-stages of zebrafish.

### Spatiotemporal expression patterns of the CYP1 family

The zebrafish CYP family 1 contains five CYP genes, i.e. CYP1A, CYP1B1, CYP1C1, CYP1C2, and CYP1D1 (GRCz11, see Table 1), which all differ in their developmental expression patterns (Goldstone et al. 2009, 2010; Jönsson et al. 2007a; Verbueken et al. 2018), tissue and organ distributions (Jönsson et al. 2007b), responses to xenobiotic inducers and inhibitors (Jönsson et al. 2007a) and catalytic activities towards xenobiotic and endogenous compounds (Scornaienchi et al. 2010a, b; Stegeman et al. 2015). This suggests that each may have distinct physiological functions and/or roles in xenobiotic biotransformation.

For all genes of the CYP1 family, transcripts have been detected whenever studied, i.e. in zebrafish from at least 3 hpf onwards (Table 2; Goldstone et al. 2010; Jönsson et al. 2007a; Verbueken et al. 2018). **CYP1A** is the only member of the CYP family 1 for which transcripts have also been detected in unfertilized zebrafish oocytes, indicating a maternal transfer of CYP1A mRNA to the embryo (Goldstone et al. 2010; Verbueken et al. 2018). The constitutive expression of CYP1A fluctuates during the first 48 hpf (Glisic et al. 2016; Goldstone et al. 2010) and considerably increases around hatching, reaching a first peak in late embryogenesis (96–120 hpf; Glisic et al. 2016; Jones et al. 2010; Verbueken et al. 2018). Controversial observations exist on CYP1A expression patterns in juvenile zebrafish, making it difficult to draw conclusions about the potential existence of differences in the extent of xenobiotic biotransformation capacities of zebrafish embryos and juveniles. While Verbueken et al. (2018) documented CYP1A to be constitutively expressed at relatively stable levels from late embryogenesis until 30 dpf, both Jönsson et al. (2007a) and Goldstone et al. (2009) documented CYP1A expression levels to fluctuate throughout embryonic and juvenile development with peaks reached at 21 dpf (Jönsson et al. 2007a) and 57 dpf (Goldstone et al. 2009). The reasons for these variable results are not obvious given that all studies used quantitative real-time PCR as method for profiling CYP1A expression in wild-type zebrafish. In zebrafish embryos at 30 hpf, CYP1A is constitutively expressed in many organs and tissues across the whole body, including eyes, heart, intestine, skin, fin bud, cloaca, intersegmental blood vessels and at lower levels also in otic vesicles and the brain (cf. Table 3; Kim et al. 2013). This is in contrast to juvenile and adult zebrafish as well as other fish species, e.g., scup (*Stenotomus chrysops;* (Stegeman et al. 1991) and turbot (*Scophthalmus maximus*; Reinecke and Segner 1998), where CYP1A is most abundantly expressed in the liver and intestine (Goldstone et al. 2009; Jönsson et al. 2007b; Taylor 2005), the major organs relevant to xenobiotic biotransformation. However, expression of CYP1A does not only vary with age, but also with sex. While CYP1A mRNA accounts for 14.5% of the total hepatic CYP mRNA contents in male zebrafish, it accounts for only 5% in female zebrafish (Kubota et al. 2019).

The constitutive expression level of **CYP1B1** increases immediately after activation of the embryonic genome, reaching a peak level in zebrafish embryos at 36 hpf (Verbueken et al. 2018). This level is not reached again in any of the later embryonic or juvenile stages studied so far (i.e. up to 30 dpf; Verbueken et al. 2018). A very similar trend, albeit with a peak in expression reached at 3 dpf and almost negligible expression levels found in juvenile zebrafish at 57 dpf, was reported by Jönsson et al. (2007a). In zebrafish embryos, beginning at 24 hpf, CYP1B1 transcripts have been localized in ocular cells (Yin et al. 2008), where maximum levels are reached between 30 and 48 hpf (Yin et al. 2008), which coincides with the start of cardiac looping (Bakkers 2011) and the onset of melanin synthesis in the retinal pigment epithelium (Glass and Dahm 2004). Moreover, CYP1B1 is expressed in the embryonic retina and midbrain–hindbrain boundary regions, but not in branchial arches, the kidney and fin buds (Yin et al. 2008). Adult zebrafish constitutively express CYP1B1 in a variety of organs with highest levels in brain, eyes and heart. Lowest levels were documented in gonads and intestine (Jönsson et al. 2007b). These spatiotemporal expression patterns led Jönsson et al. (2007b) to suggest that CYP1B1 may primarily have physiological functions in zebrafish (Jönsson et al. 2007b). However, heterologously expressed zebrafish CYP1B1 could be demonstrated to catalyze oxidative biotransformation of a number of xenobiotic compounds including *O*-alkyl derivates of resorufin and coumarin (Scornaienchi et al. 2010a, 2010b; Stegeman et al. 2015). CYP1B1 might thus also contribute to extrahepatic biotransformation processes in zebrafish.

Discrepancies exist in literature regarding the developmental expression trends of the two paralogous genes **CYP1C1** and **CYP1C2**. Jönsson et al. (2007a) documented constitutive expression levels of both CYP1C genes to fluctuate during embryonic and juvenile development, with both genes reaching minimum levels in zebrafish at 6 dpf and CYP1C2 again at 57 dpf. In contrast, in a more recent study, Verbueken et al. (2018) found expression levels of CYP1C1 and CYP1C2 to steadily increase during the first 5 to 10 dpf, then leveling off until 30 dpf. This pattern led Verbueken et al. (2018) to suggest that the biotransformation capacity might be immature during early development of zebrafish. In adult zebrafish, both genes are predominantly expressed in the brain, eyes and heart. CYP1C1 is also expressed in gills and CYP1C2 in the kidney (Jönsson et al. 2007b). Both were thus suggested to primarily have physiological functions (Jönsson et al. 2007b). However, as CYP1C1 and CYP1C2 have also been demonstrated to metabolize benzo[a]pyrene (B[a]P) to its metabolite B[a]P-7,8-diol-9,10-oxide, which involves the intermediate formation of the ultimate carcinogenic form of B[a]P (i.e. B[a]P-7,8-diol-9,10-oxide; Stegeman et al. 2015), both isoforms might also be involved in extrahepatic bioactivation processes at least in adult zebrafish.

Among the genes of the CYP1 family, the constitutive expression of **CYP1D1** peaks earliest (Goldstone et al. 2009, 2010). Its maximum expression level is reached in zebrafish embryos at 9 hpf and is two to three times higher than expression levels found in all later embryonic juvenile stages tested so far (1–7 dpf and 57 dpf; Goldstone et al. 2009). When describing the early expression peak, it has been hypothesized that CYP1D1 might have endogenous functions in early developmental processes (Goldstone et al. 2009). However, CYP1D1 has also been found to catalyze oxidative biotransformation and bioactivation of benzo[a]pyrene and a number of synthetic CYP probe substrates, although in most cases with a catalytic efficiency smaller than that of all other CYP1 isoforms (Scornaienchi et al. 2010b; Stegeman et al. 2015).

### Spatiotemporal expression patterns of the CYP2 family

The CYP2 family, by far the largest and most diverse CYP family in zebrafish, contains 42 genes (GRCz11, Table 1), all of which are being expressed from very early stages of embryonic development, i.e. from as early as 3 hpf (Goldstone et al. 2010). In addition, transcripts of **CYP2AA4** and **CYP2P6** have been found in unfertilized oocytes (Goldstone et al. 2010).

Whenever expression levels of CYP2 genes have been studied in both pre-hatch (≤ 48 hpf) and post-hatch embryonic stages (> 48 hpf), for all genes examined (i.e. **CYP2AD2**, **CYP2J26, CYP2K6, CYP2K18, CYP2N13, CYP2Y3**), except **CYP2R1** (Peng et al. 2017), higher expression levels have been documented in post-hatch stages (Jones et al. 2010; Nawaji et al. 2020; Poon et al. 2017b; Wang-Buhler et al. 2005). In the case of **CYP2K6**, transcripts were not detected in zebrafish embryos before 72 hpf, after which expression levels increased, first reaching significant levels in zebrafish embryos at 5 hpf (Wang-Buhler et al. 2005). However, up to date, no functional data are available for these isoforms, with the exception of **CYP2K6**, which was shown to catalyze the bioactivation of the mycotoxin aflatoxin B1 (Wang-Buhler et al. 2005). Thus, it remains to be clarified whether quantitative differences in CYP2 expression levels may have functional implications for the xenobiotic biotransformation and bioactivation capacities of zebrafish or might be a consequence of physiological processes.

So far, **CYP2R1** is the only CYP2 gene, whose mRNA abundance has been studied across all developmental stages (i.e. in zebrafish embryos, juveniles and adults; cf. Table 2). The constitutive expression of CYP2R1 peaks twice, once at 9 hpf and again at 28 hpf and was, therefore, suggested to have different stage-specific functions (Peng et al. 2017). One of these could be linked to vitamin D_3_ biotransformation (Peng et al. 2017). In adult zebrafish, CYP2R1 is expressed at levels much lower than those in zebrafish embryos or juvenile zebrafish (Peng et al. 2017).

Although information is available on sex-differences in constitutive expression levels of a number of CYP2 genes (cf. Table 2), nothing is yet known about the impact these differences might have on the susceptibility of female and male zebrafish to xenobiotic exposure. In a study by Kubota et al. (2013), variability between female and male zebrafish, although not significant, was noted with respect to the transcript abundance of **CYP2AA1** and **CYP2AA1** in a number of organs (e.g., liver, gonads and kidney). **CYP2J1**, which has been suggested to play a role in gonadal development and ovarian follicular development (Wang et al. 2007) and **CYP2K6** (Wang-Buhler et al. 2005) were both found to be constitutively expressed in gonads of adult zebrafish, however, at higher levels in female than in male zebrafish. Moreover, by transcriptional analysis of liver samples, Kubota et al., (2019) and Zheng et al. (2013) identified several CYP2 genes having sex-biased expression levels. These include **CYP2N13**, **CYP2K6**, **CYP2AD2** and **CYP2AA4**.

### Spatiotemporal expression patterns of the CYP3 family

The five genes of the CYP3 family, i.e. CYP3A65, CYP3C1, CYP3C2, CYP3C3 and CYP3C4, have been studied to varying extent (cf. Table 2). While development-related trends in constitutive expression levels of **CYP3A65** and **CYP3C1** were repeatedly assessed in zebrafish embryos of different ages (Chang et al. 2013; Corley-Smith et al. 2006; Glisic et al. 2016; Goldstone et al. 2010; Shaya et al. 2014; Tseng et al. 2005) and also in juvenile zebrafish up to 30 dpf (Verbueken et al. 2018), expression profiles of all other CYP3 genes have only been once determined in zebrafish older than 48 hpf (Nawaji et al. 2020).

**Fig. 2:**
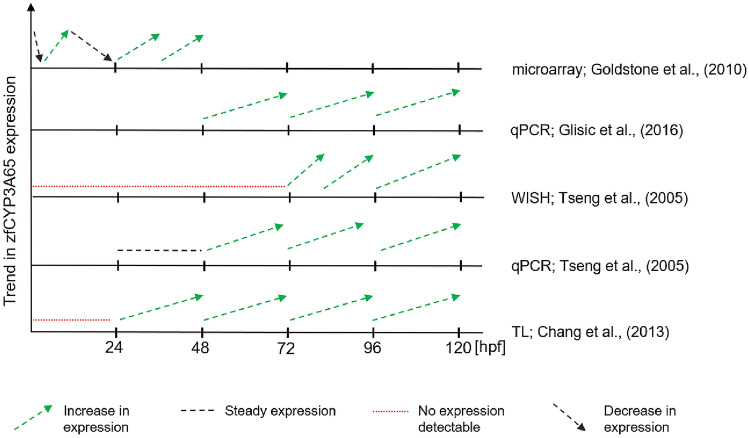
Comparison of the embryonic expression trends reported in literature for zebrafish CYP3A65. Data were generated by (**a**) microarray analysis (Goldstone et al. 2010), (**b**) qPCR (Glisic et al. 2016), (**c**) whole mount in situ hybridization (WISH; Tseng et al. 2005) and (**d**) a transgenic zebrafish line expressing CYP-eGFP constructs (TL; Chang et al. 2013)

Four different methods have been used to evaluate **CYP3A65** expression in zebrafish embryos (Fig. 2). Of these, quantitative real-time PCR (qPCR) and microarray analysis proved to be the most sensitive, allowing for detection of CYP3A65 mRNA in whole-body homogenates of zebrafish from as early as 1.5 and 3 hpf, respectively (Goldstone et al. 2010; Verbueken et al. 2018). In contrast, by means of whole mount in situ hybridization and the use of a transgenic zebrafish line expressing eGFP:CYP3A65 constructs, transcripts have not been detected until 72 and 24 hpf, respectively (Chang et al. 2013; Tseng et al. 2005).

The constitutive expression level of **CYP3A65** remains relatively low until hatching, when expression of CYP3A65 starts to increase markedly, reaching a first peak in zebrafish embryos at 120 hpf (Chang et al. 2013; Glisic et al. 2016; Goldstone et al. 2010; Nawaji et al. 2020; Tseng et al. 2005; Verbueken et al. 2018). Therefore, it might be hypothesized that biotransformation processes depending on CYP3A65 are immature during early embryonic development and thus significantly different from that of juvenile and adult zebrafish. In juvenile zebrafish, i.e. between 120 hpf and 30 dpf, the constitutive expression of CYP3A65 was documented to remain at an almost stable level slightly below the peak level measured in 120-h-old zebrafish embryos (Verbueken et al. 2018). Through immunohistochemical analyses, CYP3A65 transcripts could be localized in the corpuscle of Stannius, eyes and ears of juvenile zebrafish and, at much lower levels, in the heart, intestine and kidney (Taylor 2005; cf. Table 3). This contrasts the spatial expression patterns found in zebrafish embryos and adults, where CYP3A65 transcripts were almost exclusively restricted to liver and intestine (Tseng et al. 2005). In adult female zebrafish, CYP3A65 is the most abundantly expressed hepatic CYP isoform, making up 13.4% of the total amount of CYP mRNA. It was, therefore, suggested to play a central role in liver physiology and/or xenobiotic biotransformation (Kubota et al. 2019). However, in male zebrafish CYP3A65 mRNA accounts for only 6.5% of the total hepatic CYP mRNA content (Kubota et al. 2019). This sex-dimorphic expression is contrary to that found in adult killifish (*Fundulus heteroclitus*), where male fish displayed up to 2.5 -fold higher hepatic CYP3A65 mRNA and protein than female killifish (Hegelund and Celander 2003). Whether the sex-related differences in the hepatic mRNA abundance of CYP3A65 has consequences for the metabolic competence and/or the susceptibility of male and female zebrafish to xenobiotic-exposure remains to be clarified. By characterizing the catalytic activities of heterologously expressed CYP enzymes from zebrafish, Scornaienchi et al. (2010a) could demonstrate that CYP3A65 has activities towards compounds of both endogenous (e.g., 17β-estradiol) and exogenous origin (e.g., 7-benzyloxy-4-trifluroromethyl-coumarin), albeit with an efficiency that was much lower than that of most CYP1 isoforms (Scornaienchi et al. 2010a).

Transcripts of **CYP3C1** have been detected in zebrafish embryos from as early as the 4—8 cell stage (Shaya et al. 2014; Verbueken et al. 2018), which is prior to activation of the zygotic genome (Kane and Kimmel 1993). This indicates that CYP3C1 transcripts are maternally deposited into oocytes and might play a role in earliest developmental processes (Goldstone et al. 2010). The constitutive expression level of CYP3C1 fluctuates slightly during the first 48 h(Goldstone et al. 2010; Shaya et al. 2014) before starting to steadily increase until a peak in juvenile zebrafish at 10 dpf is reached (Verbueken et al. 2018). CYP3A65 is significantly higher expressed in male and female adult zebrafish than in zebrafish embryos and juvenile zebrafish (up to 144 hpf; Nawaji et al. 2020). The spatial distribution pattern of CYP3C1 is sex-biased and age-dependent (cf. Table 3). Transcripts of CYP3C1 are distributed throughout the whole body of 12-h-old zebrafish embryos, are concentrated in brains of 48-h-old zebrafish embryos and additionally appear in the intestine and pharynx at 120 hpf (Corley-Smith et al. 2006). In juvenile zebrafish, kidney, gills, eye, pseudobranch, and oropharynx are the major sites of CYP3C1 expression, but transcripts are also present, albeit at much lower levels, in neurons, skin, ear, taste bud, intestine and liver (Taylor 2005). In adult zebrafish, CYP3C1 is constitutively expressed in many tissues and organs, male-biased in liver, eyes and kidney, female-biased in gonads and intestine, and without significant sex-differences in brain, gills, heart, skin, spleen, and olfactory rosette (Corley-Smith et al. 2006; Shaya et al. 2014). Hence, depending on the function of CYP3C1, toxicological profiles of CYP3C1 substrates might not only vary by age, but also by sex.

Transcripts of CYP3C2 and CYP3C3 genes were detected prior to activation of the zygotic genome, i.e. from 1 and 3 hpf onwards (Goldstone et al. 2010; Shaya et al. 2014). While CYP3C2 has a bimodal expression pattern with peaks reached at 5 and 48 hpf, the constitutive expression level of CYP3C3 gradually decreases within the first hours after fertilization, eventually reaching a minimum in zebrafish embryos at 25 hpf (Shaya et al. 2014). After hatching, expression levels of CYP3C2/C3 start to markedly increase, reaching a first peak at the end of embryogenesis. Expression of CYP3C2/C3 is dependent on age and sex, with male zebrafish showing significantly higher expression levels than embryonic, juvenile (at 144 hpf) and female zebrafish (Nawaji et al. 2020). Both genes are widely expressed in several tissues and organs of adult zebrafish (e.g., brain, eyes, gills, gonads, heart, intestine, kidney, liver, olfactory rosette, and spleen; cf. Table 3), with brain, eyes and heart showing female-biased expression levels (Shaya et al. 2014).

Expression of CYP3C4 fluctuates during embryogenesis and reaches a peak level in juvenile zebrafish at 144 hpf. This peak level was documented to be approximately double that of adult female and four times that of male zebrafish (Nawaji et al. 2020). In adult zebrafish, CYP3C4 transcripts were documented in several organs and tissues, with highest levels found in brain, eyes and intestine of male zebrafish and gills, gonads and kidney of female zebrafish (Shaya et al. 2014).

### Spatiotemporal expression patterns of the CYP4 family

The zebrafish CYP4 family contains four genes, i.e. **CYP4F3, CYP4T8, CYP4V7 and CYP4V8** (GRCz11, see Tab 1). For none of these, information on potential roles in xenobiotic biotransformation is available. Moreover, information on developmental expression patterns of the CYP4 genes is currently limited to zebrafish embryos ≤ 48 hpf. Although transcripts of all CYP4 genes have been detected in zebrafish at 3 hpf (Goldstone et al. 2010), and for all except CYP4V8, expression peaks are reached within the first 6 hpf, no conclusions can be drawn with regard to the development of CYP4-dependent biotransformation capacities in zebrafish.

## Activities towards synthetic CYP probe substrates

### Metabolism of cytochrome P450 probe substrates

Given the complexity of the CYP system, a single assay or probe substrate appears to be hardly sufficient to assess the CYP-dependent biotransformation capacity of organisms. Instead, a comprehensive testing strategy covering a range of CYP activity assays is needed. In fact, during the past two decades, a variety of routinely used fluorescence- and luminescence-based assays for assessing CYP activities in mammals have been modified for in vitro and in vivo use in fish species, e.g., common carp (*Cyprinus carpio;* Funari et al. 1987), fathead minnow (*Pimephales promela;,* Boehler et al. 2018), guppy (*Poecilia reticulata;* Funari et al. 1987), medaka (*Oryzias latipes*, Funari et al. 1987), rainbow trout (*Oncorhynchus mykiss;* Andersson and Goksøyr 1994; Burkina et al. 2018; Smith 2009)*,* killifish (*Fundulus heteroclitus*; Smith 2009) and zebrafish *(Danio rerio*; Chng 2013; Gonzalez-Doncel et al. 2011; Jönsson et al. 2009; Otte et al. 2017; Verbueken et al. 2017; see Tables. 4, 5). These assays rely on the use of synthetic pro-luciferin acetals and non- or low-fluorescent *O*-alkyl derivates of coumarin, fluorescein, quinoline and resorufin, which are metabolized by CYP enzymes into active luciferin (Cali et al. 2006) and highly fluorescent products, respectively. Probe substrates that have been used in zebrafish are listed in Table 4. Included among these are specific (e.g., luciferin isopropyl acetal; Doshi and Li (2011) and selective (e.g., 7-benzyloxyresorufin and 7-methoxy-4-trifluorormethylcoumarin; Pastrakuljic et al. 1997; Stresser et al. 2002) probe substrates of mammalian CYP1, CYP2 and CYP3 isoforms. Yet, there are no studies investigating CYP activities in (zebra)fish by using mammalian CYP4 probe substrates such as luciferin-4A or lauric acid (Yamaori et al. 2018).

**Table 4:** Spatiotemporal patterns of CYP-dependent activities in embryonic, juvenile and adult stages of zebrafish (*Danio rerio*)

Activity assay	Zebrafish CYPs	Embryo		Juvenile		Adult		Method	References
In vivo* 7-*benzyloxy-4-trifluroromethyl-*O*-debenzylase (BFCOD) assay	CYP1A > CYP1C2 > CYP1C1 = CYP1B1 > CYP3A65	4 dpf	Constitutive activity				0–4 dpf; 28 °C/27 °C; 100 µM; spectrofluorophotometer,microplate reader; kinetic measurement		Creusot et al. (2015)
In vivo* 7-*benzyloxy-4-trifluroromethyl-*O*-debenzylase (BFCOD) assay	CYP1A > CYP1C2 > CYP1C1 = CYP1B1 > CYP3A65	120 hpf	Constitutive activity	122 hpf, 9 dpf	Activity above the limit of quantification		24–120 hpf; 28 °C; 20 µg/L; epifluorescence microscope		Oziolor et al. (2017)
In vivo 7-benzyloxy-methyl-resorufin-*O*-debenzylase (BOMR) assay	n.s	7—50 hpf	Activity below the limit of quantification				60 min; 28.5 °C; 4 µM; fluorescence microscope		Verbueken et al. (2018)
		74 hpf	Activity above the limit of quantification						
		98 hpf	Peak in activity	14 dpf	Activity below the limit of quantification				
In vitro 7-benzyloxy-methyl-resorufin-*O*-debenzylase (BOMR) assay	n.s	5—120 hpf	Activity only observed at 72 and 96 hpf Activity close to the limit of quantification				Whole-body homogenates; 60 min; 28 °C; 1.2 µM; spectrofluorophotometer, microplate reader, kinetic measurement		Verbueken et al. (2017)
In vitro 7-benzyloxy-methyl-resorufin-*O*-debenzylase (BOMR) assay	n.s	5—48 hpf	Activity below the limit of quantification	9 dpf	Activity below the lower limit of quantification	Whole body microsomes: 1.34 ± 0.51 pmol/min/mg MP Activity significantly higher than in microsomes of all earlier stages	Whole-body microsomes; 72 min; 28 °C; 1.2 µM; spectrofluorophotometer, microplate reader, kinetic measurement		Verbueken et al. (2018)
		72 hpf	0.36 ± 0.35 pmol/min/mg MP	14 dpf	Activity 0.64 ± 0.09 mol/min/ mg MP				
		96 hpf	0.29 ± 0.13 pmol/min/mg MP						
		120 hpf	Activity above the lower limit of quantification						
In vitro 7-benzyloxy-resorufin-*O*-debenzylase (BROD) assay	CYP1A = CYP1B1 > CYP1Cs > CYP1D1	2.5—96 hpf	Activity below the limit of quantification				S9 fraction of refined preparation; 60 min; 37 °C; 5 µM; spectrofluorophotometer, microplate reader		Otte et al. (2017)
		120 hpf	Activity above the limit of detection, but below limit of quantification						
In vivo 7-ethoxy-coumarin-*O*-dealkylase (ECOD) assay	n.s	96 hpf	Constitutive activity					Up to 10 h; 28 ± 1 °C; 100 µM; fluorimeter	Jones et al. (2010)
In vitro 7-ethoxy-coumarin-*O*-dealkylase (ECOD) assay	n.s				Constitutive activity: liver > gill > muscle > brain; significant inducibility			Microsomes; 30 min; 30 °C; ~ 33 µM; spectrofluorophotometer, microplate reader	Wu et al. (2014)
In vitro 7-ethoxy-coumarin-*O*-dealkylase (ECOD) assay	n.s					Liver microsomes: constitutive activity		Liver microsomes; 30 min; 20 – 22 °C; 0.3 µM; spectrofluorophotometer	Funari et al. (1987)
In vivo 7-ethoxy-resorufin-*O*-deethylase (EROD) assay	CYP1A > CYP1C2 > CYP1B1 = CYP1C1 > CYP1D1	8 hpf	Cytoplasm of the cells of the envelope layer, yolk syncytial layer and developing germ layers	128 hpf	Same spatial distribution as at 104 hpf			10 min; 20 °C; 0.4 µg/ml, confocal laser scanning microscope	Otte et al. (2010)
		32 hpf	Head: 4^th^ ventricle, tectorial ventricle, otic vesicle, hyoid mesenchyme, telencephalon/olfactory placode mandibular mesenchyme Trunk: straight tube of the heart, dorsal aorta, myotomes, envelope of the yolk, pronephric duct, urogenital pore Circulatory system: vessels of the brain, aortic arches, dorsal aorta, axial vein, pericardium, heart						
		56 hpf	Head: inner parts of the eye, 4^th^ ventricle, mesencephalon						
		104 hpf	Trunk: primordia of the kidney, pronephric duct, urogenital pore, liver primordium Trunk: intestine, liver, anal pore, head kidney, nephric duct, kidney, urinary pore Circulatory system: inner optic circle, vessels in the eye background and the brain, branchial arches, heart, rete mirabile, vascular tissue surrounding the yolk sax, etc.						
In vivo 7-ethoxy-resorufin-*O*-deethylase (EROD) assay	CYP1A > CYP1C2 > CYP1B1 = CYP1C1 > CYP1D1	7 hpf	Blastoderm, germ ring Significant higher activity than in all later developmental stages	122 hpf	Intestine			60 min; 28.5 °C; 1.7 µM; fluorescence microscope	Verbueken et al. (2018)
		26 hpf	Whole embryo, hatching gland	14 dpf	No resorufin formation detected				
		50 hpf	Whole embryo, otic vesicle						
		74 hpf	Intestine, liver, otic vesicle						
		98 hpf	Intestine, liver, pronephric duct						
In vitro 7-ethoxy-resorufin-*O*-deethylase (EROD) assay	CYP1A > CYP1C2 > CYP1B1 = CYP1C1 > CYP1D1	2.5 hpf	Activity above the limit of detection but lower than the limit of quantification					S9 fraction of refined preparation; 60 min; 37 °C; 5 µM; spectrofluorophotometer, microplate reader	Otte et al. (2017)
		48 hpf	Activity below the limit of detection						
		96—120 hpf	Activity above the limit of quantification						
In vitro 7-ethoxy-resorufin-*O*-deethylase (EROD) assay	CYP1A > CYP1C2 > CYP1B1 = CYP1C1 > CYP1D1	5 hpf	Highest activity, large inter-batch variation: 1.50 ± 1.40 pmol RS/mg/min			Liver microsomes: much higher activity than in embryonic stages, no gender differences		Whole-body and liver microsomes; up to 2 h; 28.5 °C; 10 µM; spectrofluorophotometer, microplate reader (kinetic measurement)	Saad et al. (2016b)
		24—48 hpf	Negligible activity 0.33 ± 0.29 and 0.14 ± 0.15 pmol RS/mg/min						
		72 -96 hpf	Increase in activity, even further at the end of organogenesis 0.60 ± 0.50 and 0.91 ± 0.47 pmol RS/mg/min						
		120 hpf	Negligible activity 0.31 ± 0.20 p mol RS/mg/min						
In vitro 7-ethoxy-resorufin-*O*-deethylase (EROD) assay	CYP1A > CYP1C2 > CYP1B1 = CYP1C1	8—104 hpf	Peak in activity at 8 hpf and 104 hpf Minimum activity at 32 hpf	128 hpf	Activity 50 – 30% of the level at 104 hpf			Whole-body microsomes; 20 min; 20 °C; ~ 1 µM; spectrofluorophotometer, microplate reader	Otte et al. (2010)
In vitro 7-ethoxy-resorufin-*O*-deethylase (EROD) assay	CYP1A > CYP1C2 > CYP1B1 = CYP1C1 > CYP1D1			2 wpf	Constitutive and significant inducible activity			Whole-body microsomes; spectrofluorophotometer	Pauka et al. (2011)
In vitro 7-ethoxy-resorufin-*O*-deethylase (EROD) assay	CYP1A > CYP1C2 > CYP1B1 = CYP1C1 > CYP1D1					Whole gill arches and liver microsomes: Constitutive and significant inducible activity		Whole gill arches/liver microsomes; 10 min, 30 min/10 min; 20 °C; 1 µM / ~ 10 µM; spectrofluorophotometer, microplate reader	Jönsson et al. (2009)
In vivo 7-methoxy-coumarin-*O*-demethylase *(*MCOD) assay		5.5 hpf	Cytoplasm of the cells of the envelope layer					120 min; 26 ± 1 °C; 1 mM; confocal laser scanning microscope	Loerracher et al. (2020)
	n.s	12 hpf	Entire embryonic body						
		24-48 hpf	Brain ventricles, cardiovascular system						
		56 hpf	Cardiovascular system, urinary tract (i.e. pronephros), intestine						
		72—118hpf	Cardiovascular system: aortic arches, common cardinal vein plexus, dorsal aorta, dorsal longitudinal anastomosing vessels, intersegmental blood vessels, vascular tissue of the yolk sac, vessels of the brain and head Gastrointestinal tract: intestine, liver, pancreas Urinary tract: pronephros, pronephric duct					120 min; 26 ± 1 °C; 1 mM; confocal laser scanning microscope	
In vivo* n*-octyloxy-methyl-resorufin-*O*-dealkylase (OOMR) assay	n.s	96 hpf	Constitutive activity					≤ 10 h; 28 ± 1 °C; 8 µM; fluorometer	Jones et al. (2010)
In vitro 7-pentoxy-resorufin-O-depentylase (PROD) assay	CYP1A > CYP1C1 > CYP1C2 > CYP1D1	2.5—120 hpf	No activity above limit of detection					S9 fraction of refined preparation; 60 min; 37 °C; 5 µM; spectrofluorophotometer, microplate reader	Otte et al. (2017)
In vivo luminescence-based Promega P450-Glo™ CYP3A4 assay (luciferin-6’-benzylether)		48 hpf	Constitutive activity					30 min; 37 °C; 50 µM; spectrofluorophotometer, microplate reader	Li et al. (2011)
	n.s	72 hpf	Constitutive activity higher than at 48 hpf						
In vivo luminescence-based Promega P450-Glo™ CYP3A4 assay (luciferin isopropyl acetal (luciferin-IPA), luciferin-6’-pentafluoro-benzyl ether (luciferin-PFBE))	n.s	120 hpf	Constitutive activity					240 min/60 min; 28.5 °C; Luciferin-PFBE: 5, 25, 100, 200, 300, and 500 µM; Luciferin-IPA: 0.3, 3, 15 and 30 µM; spectrofluorophotometer, microplate reader	Chng (2013)
In vitro luminescence-based Promega P450-Glo™ CYP3A4 assay (luciferin isopropyl acetal)	n.s					Liver microsomes: Activity lower than the limit of quantification		Liver microsomes; 10 min; 37.5 °C; 4 µM;	Verbueken et al. (2017)

*MC* microsomal protein, *MP* microsomal protein, *n.s.* not specified, *RS* resorufin

Due to the limited knowledge regarding isoform specificity of mammalian CYP probe substrates in (zebra)fish (Scornaienchi et al. 2010b), the interpretation of fluorogenic and luminogenic CYP-activity assays remains challenging. An additional complicating factor is the characterization of several CYP isoforms by broad and overlapping substrate specificities (Scornaienchi et al. 2010b; Stegeman et al. 2015). Many CYPs are capable of metabolizing biotransformation reactions of different xenobiotic compounds that are not necessarily structurally related, and, vice versa, many xenobiotic compounds are metabolized in the same way by different CYP isoforms, albeit often with distinct efficiencies. For instance, heterologously expressed zebrafish CYP1B1, CYP1C1, CYP1C2 and CYP3A65 isoforms, and in particular the isoform CYP1A, have been found to be active in oxidative biotransformation (i.e. *O-*dealkylation) of 7-benzyloxy-4-(trifluoromethyl)-coumarin (Scornaienchi et al. 2010a, b), which is a selective, but not specific human CYP3A4 probe substrate (Stresser et al. 2002).

**Fig. 3:**
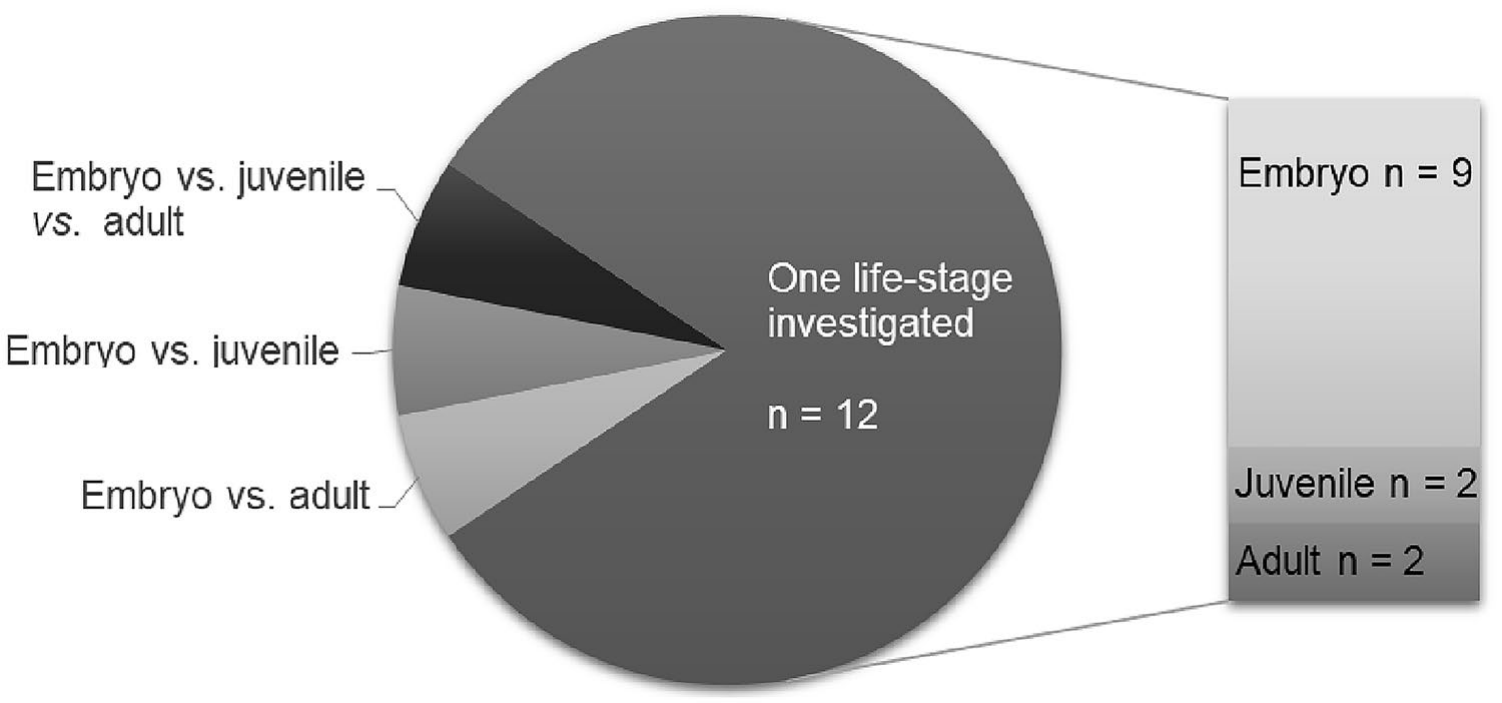
Number of studies characterizing CYP-dependent activities by fluorescent or luminescent-based assays in embryonic, juvenile and adult life-stages of zebrafish (*Danio rerio)*

Not all studies provide precise information on the experimental conditions used (e.g., ‘room temperature’, no information on the probe substrate concentration; Tables 4, 5), thus rendering reproducibility and comparison between studies difficult. When experimental conditions were reported, these varied considerably: incubation time ranged from 10 min to 24 h, temperature from 20 to 37 °C and probe substrate concentration from 0.3 µM to 1 mM. Activities towards CYP probe substrates were either assessed dynamically, i.e. by measuring increases in fluorescence over time, or statically by measuring intensities of fluorescence or luminescence at a single time-point. In cases where embryonic or juvenile zebrafish were used, CYP activities were monitored both in vivo and in vitro using living organisms, subcellular fractions (e.g., S9 fractions and microsomes) prepared from whole-body homogenates or subcellular fractions prepared from different tissues and organs, such as liver, gills, muscles and brain. In cases where adult zebrafish were used, four out of five studies used microsomes prepared from liver samples, one used whole gill arches and microsomes prepared from liver samples, and one used microsomes prepared from whole-body homogenates. To date, most studies have focused on investigating the level of CYP activity in one developmental stage or even at a single point in development (Fig. 3). To the best of our knowledge, up to now, four studies are available comparing CYP activity levels across different developmental stages of zebrafish (i.e. embryo *vs.* juvenile and embryo *vs.* adult). Out of these, only one has systemically assessed and compared CYP activity levels across all developmental stages. In this study, published by Verbueken et al. (2018), benzyloxymethylresorufin-*O*-deethylase (BOMR) activities were assessed in microsomes prepared from whole-body homogenates of embryonic, juvenile and adult zebrafish. Furthermore, the study by Verbueken et al. (2018) demonstrated a substantial risk of underestimating CYP (i.e. BOMR) activities when directly comparing activity levels of zebrafish whole-body microsomes with those of zebrafish liver microsomes, as has been done in most previous studies comparing CYP activity levels between embryonic and adult zebrafish (e.g., Saad et al. 2016b; Verbueken et al. 2017). Hence, allowing for conclusions to be drawn concerning potential developmental-related differences in CYP activity levels, there is an urgent need for more systematic approaches as the one chosen by Verbueken et al. (2018).

**Table 5:** Spatiotemporal patterns of CYP-dependent activities in ecotoxicologically relevant model fish species

Fish species	Activity assay Probe substrate	Embryo	Juvenile	Adult	Method	References
Atlantic salmon (*Salmon salar*)	**In vitro fluorogenic CYP activity assays** **Probe substrates:** 7-benzyloxyresorufin, 7-ethoxyresorufin, 7-benzyloxy-4-trifluoromethyl-coumarin, 7-benzyloxyquinoline			Constitutive activities towards all tested probe substrate Differences in responses to ketoconazole compared to pigs	Liver microsomes from one male and three females); 5—10 min; 25 °C; 2—20 µM; HPLC	Zlabek and Zamaratskaia (2012)
Fathead minnow (*Pimephales promelas)*	**In vivo 7-ethoxy-resorufin-*O*-deethylase (EROD) assay** **Probe substrate:** 7-ethoxyresorufin	120 hpf: constitutive and inducible activity in the gastrointestinal tract			20 min; 26 ± 1 °C; 0.1 mg/L; epifluorescence microscope	Boehler et al. (2018)
Medaka *(Oryzias latipes)*	**In vitro fluorogenic CYP activity assays** **Probe substrates:** dibenzylfluorescein, 7-ethoxyresorufin			Constitutive and xenobiotic-inducible activities towards both probe substrates	Liver microsomes;15 min/20 min + 120 min;25 °C; 1 µM/2 µM; microplate reader	Lin et al. (2014)
Mummichog killifish (*Fundulus heteroclitus)*	**In vitro fluorogenic CYP activity assays** **Probe substrates:** 7-ethoxyresorufin; 7-benzyloxyresorufin; 7-methoxyresorufin; 7-pentoxyresorufin, 3-cyano-7-thoxycoumarin, 7-methoxy-4-amino-methyl-coumarin, 3-[2-(*N*,*N*-diethyl-*N*-methylammonium)ethyl]-7-methoxy-4 methyl-coumarin, 7-benzyloxy-4-trifluoromethyl-coumarin, 7-benzyloxyquinoline, dibenzylfluorescein			Significant higher constitutive activities towards all tested substrates than measured in liver microsomes of juvenile rainbow trout, except for 7-benzyloxyquinoline and 7-benzyloxy-4-trifluoromethyl-coumarin Significant differences in constitutive 7-ethoxyresorufin-*O*-dealkylase and 7-methoxyresorufin-O-demethylase activities between male and female fish	Liver microsomes; 10 min; 20 °C; 2—1000 µM; spectrofluorophotometer, microplate reader (kinetic measurement)	Smith and Wilson (2010)
Rainbow trout (*Oncorhynchus mykiss)*	**In vitro 7-ethoxy-resorufin-*O*-deethylase (EROD) assay**			Highest activity in the olfactory bulb; evenly distributed between telencephalon, optic tectum, hypothalamus and cerebellum	Supernatants of different brain homogenates; ≤ 20 min; 20 °C; fluorometer	Andersson and Goksøyr (1994)
Rainbow trout (*Oncorhynchus mykiss*)	**In vitro 7-ethoxy-resorufin-*O*-deethylase (EROD) and *7-*benzyloxy-4-trifluroromethyl-*O*-debenzylase (BFCOD) assay**		Constitutive EROD and BFCOD activities varied across 8 fish from 8.3 to 53.3 pmol/min/mg and from 180 to 64 pmol/min/ mg, respectively		Liver microsomes; ≤ 5 min; 5/10 min; 2/20 µM; HPLC	Burkina et al. (2018)
Rainbow trout (*Oncorhynchus mykiss*)	**In vitro 7-ethoxy-resorufin-*O*-deethylase (EROD) assay**		Induction of EROD activity in gills, but not in kidney and liver of juvenile fish upon environmental exposure		Gill filaments, kidney and liver microsomes); 10 min; 12, 21 °C; 1 µM; fluorescamine-based assay, multi-well plate reader	Abrahamson et al. (2007)
Gilthead seabream (*Sparus aurata*)	**In vitro 7-ethoxy-resorufin-*O*-deethylase (EROD) assay**		Immature males: similarities and differences in time-, concentration- and inducer-dependent EROD responses between gills, kidney and liver Highest constitutive and induced EROD activities in liver		Microsomes from gills, kidney and liver; 20 °C; 0.4 µM; multi-well plate fluorimeter	Ortiz-Delgado et al. (2008)

### Mammalian CYP1-like activities

The 7-ethoxyresorufin-*O-*deethylase (EROD) activity assay is the most commonly used method for analyzing the presence of CYP1 activities in vertebrates (De Almeida et al. 2011; Parente et al. 2008; Whyte et al. 2000). In zebrafish, all five enzymes of the CYP1 family (i.e. CYP1A, CYP1B1, CYP1C1, CYP1C2 and CYP1D1) are principally capable of *O*-deethylating 7-ethoxyresorufin (ER). Of these, CYP1A has the highest catalytic efficiency, which is 1–4 orders of magnitude higher than that of all other zebrafish CYP1s (Scornaienchi et al. 2010a, 2010b). In vitro and in vivo studies have documented the presence of EROD activity from as early as the blastula (i.e. 2.5 and 5 hpf; Otte et al. 2017; Saad et al. 2016a, b) and the gastrula stage (i.e. 7 and 8 hpf; Otte et al. 2010; Verbueken et al. 2018). To our knowledge, the documentation of EROD activity happened much earlier in zebrafish than in other fish species (Table 4 vs. Table 5). So far, the earliest report of EROD activity in fathead minnow and medaka is at 144 hpf (Boehler et al. 2018) and 2 dpf (Gonzalez-Doncel et al. 2011), respectively. During zebrafish embryogenesis, the whole-body EROD activity peaks within the first 10 h of embryonic development, then decreases to a minimum reached in the pharyngula period and subsequently increases again around the time of hatching (Otte et al. 2010; Saad et al. 2016b; Verbueken et al. 2018). The early peak in EROD activity has been suggested to be a consequence of maternal CYP mRNA transfer (Saad et al. 2016a, b). At the end of embryogenesis, EROD activity tends to decrease again as indicated by EROD activities measured in early juvenile, which were lower rather than in embryonic stages of zebrafish (Otte et al. 2010; Saad et al. 2016a, b; Verbueken et al. 2018). The extent to which juvenile zebrafish possess EROD activities is still not clear, as the two studies currently available in literature show inconsistent results: While Pauka et al. (2011) documented constitutive and significantly inducible EROD activities in subcellular fractions prepared from whole-body homogenates of 2-weeks-old zebrafish, a more recent study published by Verbueken et al. (2018) could not detect EROD activity by epifluorescence microscopy in juvenile zebrafish at 14 dpf (Verbueken et al. 2018). The ability of adult zebrafish to *O*-deethylate ER and, thus, the constitutive presence of CYP1-dependent activity has been confirmed in vitro using liver microsomes and whole gill arches (Jönsson et al. 2009; Saad et al. 2016b). However, owing to differences in sample preparation and data normalization (i.e. resorufin gill arch^−1^ min^−1^ *vs*. resorufin × mg protein^−1^ × min^−1^), the actual values of hepatic and gill EROD activities cannot be compared to each other directly. Although in other fish species, such as killifish (Smith and Wilson 2010), the level of hepatic EROD activity was documented to depend on sex, this could not be confirmed in zebrafish (Saad et al. 2016a, b).

### Mammalian CYP2-like activities

The fluorescent probe substrates 7-benzyloxyresorufin (BR), 7-ethoxycoumarin (EC), 7-methoxycoumarin (MC) and 7-pentoxyresorufin (PR) have all been used to monitor mammalian CYP2-like activities in zebrafish (cf. Table 4). However, since both resorufin derivates have been demonstrated to undergo *O-*dealkylation catalyzed by heterologously expressed zebrafish CYP1A, CYP1C1, CYP1C2 and CYP1C2 enzymes, and in addition BR also by CYP1B1 (Scornaienchi et al. 2010b), at least BR and PR might not be specific for monitoring activities of CYP2 isoforms in zebrafish. Up to now, there are no data available as to which CYP isoforms are active in catalyzing *O*-dealkylation of EC and MC in zebrafish.

**Fig. 4:**
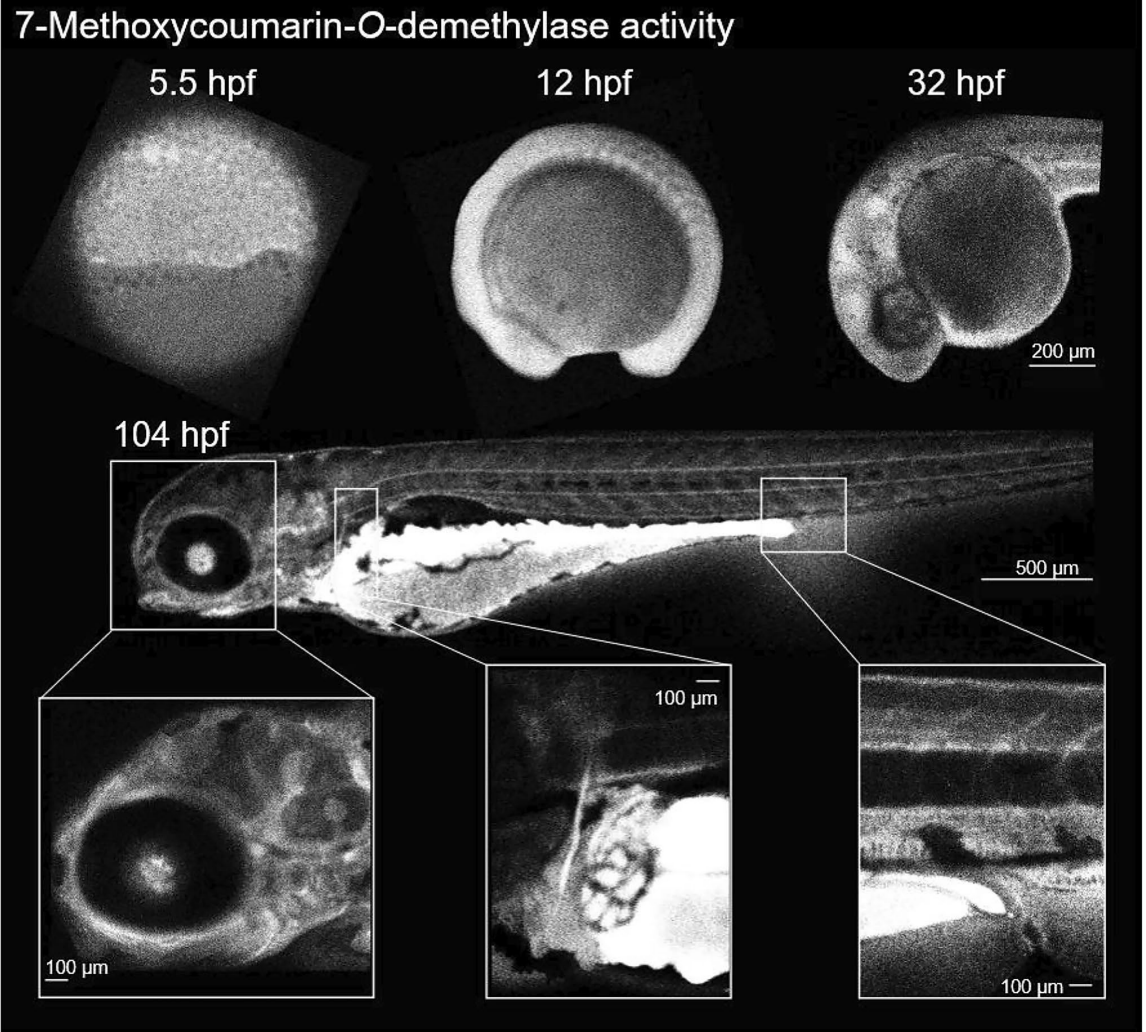
Developmental pattern of 7-methoxycoumarin-*O-*demethylase (MCOD) activity in zebrafish (*Danio rerio)*. Lateral views of zebrafish embryos exposed to 1 mM 7-methoxycoumarin for 3 h

By monitoring *O*-dealkylation of 7-methoxycoumarin (i.e. formation of 7-hydroxycoumarin), Loerracher et al. (2020) could document that zebrafish embryos exhibit mammalian CYP2-like activities from as early as 5.5 hpf. This was much earlier than expected from all previous studies. In the same study, the use of a confocal laser scanning microscope allowed for detailed visualizing the developmental pattern of 7-methoxycoumarin-*O-*demethylase (MCOD) activity throughout zebrafish embryogenesis (Fig. 4).

Otte et al. (2017) examined 7-benzyloxyresorufin-*O*-debenzylase (BROD) and 7-pentoxyresorufin-*O*-depentylase (PROD) activities in whole-body microsomes of different embryonic stages starting at 2.5 hpf. However, for both substrates, the rates of resorufin formation remained below the limit of detection (i.e. 0.7–1.3 fmol resorufin/min/embryo) until 120 hpf, when BROD activity became detectable, but could still not be quantified (< 1.4–2.6 fmol resorufin/min/embryo; Otte et al. 2017). Jones et al. (2010) documented 96 h old zebrafish embryos to metabolize 7-ethoxycoumarin and to excrete the metabolite, 7-hydroxycoumarin, into the surrounding medium. Earlier embryonic stages were not investigated in their study.

Up to now, 7-ethoxycoumarin is the only CYP2 probe substrate that has been used to monitor mammalian CYP2-like activities in juvenile or adult zebrafish. In juvenile zebrafish, 7-ethoxycoumarin-*O*-deethylase (ECOD) activity was investigated in microsomal fractions prepared from different tissues and organs. Of all tissues tested (i.e. liver, muscle and brain), the liver showed the highest constitutive level of ECOD activity (Wu et al. 2014). Funari et al. (1987) assessed ECOD activities in liver microsomes of adult zebrafish and found the levels to be comparable to those of rainbow trout (*Oncorhynchus mykiss*), common carp (*Cyprinus carpio*) and bluegill sunfish (*Lepomis macrochirus*).

### Mammalian CYP3-like activities

The pro-fluorescent and pro-luminescent CYP probe substrates 7-benzyloxy-4-(trifluoromethyl) coumarin (BFC), *N*-ocytoxymethylresorufin (OOMR), luciferin-6’-benzylether (luciferin-BE), luciferin-6’-pentafluorobenzyl ether (luciferin-PFBE) and luciferin-isopropyl acetal (luciferin-IPA) are all selective, if not specific for human CYP3A isoforms (Cali et al. 2006, 2009; Renwick et al. 2000). Studies on these substrates allowed for conclusions with regard to the presence of mammalian CYP3A-like activities in zebrafish, which is of great significance considering the central role of the human orthologue CYP3A4 in biotransformation of pharmaceuticals (Wienkers & Heath, 2005). However, for most of these probe substrates, the isoform specificity has not yet been clarified in zebrafish. An exception is BFC, which was shown to undergo *O*-dealkylation by heterologously expressed CYP1A, CYP1B1, CYP1C1 and CYP1C2 enzymes (Scornaienchi et al. 2010b). One clear gap, as becomes evident from Table 4, is the lack of studies investigating the presence of mammalian CYP3-like activities in zebrafish embryos < 48 hpf. Moreover, there is only one study available that yields information about mammalian CYP3-like activity levels at different points in zebrafish development. In the study published by Li et al. (2011), the authors demonstrate 3-day-old zebrafish embryos to have higher, although not statistically significantly higher constitutive activities towards the luminogenic probe substrates luciferin-BE, when compared with two days old zebrafish embryos. Jones et al. (2010) used the probe substrate OOMR to study mammalian-like CYP3 activities in 96-h-old zebrafish embryos and demonstrated their ability to metabolize OOMR and to excrete its metabolite 7-hydroxyresorufin into the surrounding medium. Another in vivo study conducted by Chng (2013) provided evidence that 120-h-old zebrafish embryos possess both constitutive and inducible mammalian CYP3-like activities. To date, however, no study has been conducted to examine the level or presence of mammalian CYP3-like activities in juvenile zebrafish (cf. Table 4), and the only study that evaluated the presence of mammalian CYP3-like activities in adult zebrafish by monitoring the turn-over of luciferin-IPA to D-luciferin did not find any activity above the limit of detection (Verbueken et al. 2017).

### Current state of knowledge on CYP gene expression patterns vs. CYP activities

Yet, our functional understanding of the zebrafish CYP system is far better than that at the gene expression level. Some reasons for this may include the current lack of knowledge about the CYP isoform specificity of mammalian CYP probe substrates in zebrafish (Scornaienchi et al. 2010b), the complexity of data interpretation and the missing standardization, which hinder comparison of data across different studies. Although zebrafish embryos of different ages have been documented to biotransform fluorogenic and luminogenic CYP1, CYP2 and CYP3 probe substrates in a way similar to mammals, the lack of data for juvenile and adult zebrafish hinders quantitative conclusions concerning potential age-related differences in functional biotransformation activities of zebrafish.

## Bioactivation of pro-toxicants and pro-teratogens

Another common approach to assess the functionality of the CYP system in (zebra)fish has been monitoring effects of pro-teratogens and pro-toxicants in organisms. However, this indirect way of assessing biotransformation activities faces one big challenge: How to distinguish between direct effects of pro-teratogens and pro-toxicants, and those (i.e. indirect) mediated by their bioactivated metabolites? Admittedly, while for mammals, for instance, it is known that that several pro-teratogens and pro-toxicants (e.g., aflatoxin B_1_, carbamazepine, cyclophosphamide and phenytoin) need to undergo CYP-mediated bioactivation prior eliciting their ultimate toxic or teratogenic effects (Dohnal et al. 2014; Hill et al. 2010), corresponding information is lacking for zebrafish. Hence, today, we still rely on read-across, i.e. utilization of mechanistic information gained from studies in mammals, and extrapolation.

An example of such a read-across approach is provided by the study of Klüver et al. (2014), who investigated the acute toxicity of allyl alcohol in zebrafish at different ages. So far, allyl alcohol is the only pro-toxicant known to be less toxic to zebrafish embryos than to juvenile or adult zebrafish due to lack of bioactivation in embryos (Klüver et al. 2014). In mammals, allyl alcohol is biotransformed via oxidation into acrolein, a reactive toxic aldehyde metabolite (Auerbach et al. 2008; Ohno et al. 1985). Based on read-across, in zebrafish embryos the reduced toxicity of allyl alcohol could be documented to be caused by a lack of the alcohol dehydrogenase 8a enzyme (Klüver et al. 2014).

Concerning pro-teratogens known to undergo CYP-mediated bioactivation, at least in mammals there is ample evidence, but not yet conclusive proof that zebrafish embryos possess sufficient biotransformation capacities to bioactivate these compounds to a meaningful extent. Although Weigt et al. (2011) demonstrated that ten well-known mammalian pro-teratogens, with eight of them being pharmaceuticals (carbamazepine, phenytoin, trimethadione, cyclophosphamide, ifosfamide, tegafur, and thio-TEPA) were teratogenic to zebrafish embryos when exposed for 3 days (Weigt et al. 2011), it has not yet been demonstrated that bioactivated metabolites were actually formed. Furthermore, it cannot be ruled out that even the parent compounds themselves might be teratogenic to zebrafish embryos.

## Xenobiotic metabolite profiles in zebrafish

Over the past ten years, there was a clear upward trend in the number of studies assessing biotransformation activities in zebrafish by monitoring formation of phase I and phase II metabolites. These studies provide conclusive evidence that zebrafish at different developmental stages do have metabolic competencies to perform several types of phase I and phase II biotransformation reactions (Table 6). These include inter-aromatic hydroxylation (Alderton et al. 2010; Brox et al. 2016b; Chng et al. 2012; Poon et al. 2017a, b; Saad et al. 2017; Zindler et al. 2020), *N*-dealkylation (Alderton et al. 2010; Saad et al. 2017), *O*-dealkylation (Alderton et al. 2010; Saad et al. 2017), glucuronide conjugation (Alderton et al. 2010; Kantae et al. 2016; Le Fol et al. 2017b), sulfation (Brox et al. 2016a, b; Kantae et al. 2016; Le Fol et al. 2017b; Li et al. 2015) and *O-*acylation (Zindler et al. 2020). Whole xenobiotic metabolite spectra, however, were only rarely determined (e.g., by Brox et al. 2016a; Zindler et al. 2020), probably because such studies are particularly demanding in terms of expertise, resources and funding. Moreover, as most studies today have focused on late embryonic stages of zebrafish (i.e, 72–120 hpf; e.g., Alderton et al. 2010; Jones et al. 2012; Kantae et al. 2016; Le Fol et al. 2017a; Zindler et al. 2020) or adult zebrafish (e.g., Chng et al. 2012; Li et al. 2015; Poon et al. 2017a, b; Saad et al. 2018; Wang et al. 2016), vital information concerning metabolic activities in early embryonic and juvenile stages is currently lacking. Therefore, it remains necessary to explore from which developmental stages certain biotransformation pathways are present and sufficiently developed to biotransform and bioactivate xenobiotic compounds to a biologically relevant extent.

**Table 6:** Metabolic profiles of xenobiotics in embryonic, juvenile and adult stages of zebrafish (Danio rerio)

Substance	Embryo	Juvenile	Adult	Method	Reference
1-Chloro-2,4-dinitrobenzene (CDNB)	4, 26 and 74 hpf Phase II glutathione conjugate detected in all samples exposed to CDNB for longer than 2 h Lowest concentration in ZF embryos at 4 hpf No differences in the concentration of the glutathione conjugate between ZF embryo and ZF larvae when exposed for more than 2 h *N*-acetylcysteine-*S*-conjugate detected in all embryos exposed to CDNB. Higher concentration in ZF larvae than in ZF embryos			In vivo; 24 h 0.12 µM (0.01% ethanol) LC-HRMS	Tierbach et al. (2020)
Acetaminophen (Paracetamol)	3 dpf Phase II sulfate and glucuronide metabolites identified Amounts excreted relatively low compared to amount in larvae			In vivo; 1 h + 1–4 h drug-free medium; 1 mM; UPLC/QTOF/MS	Kantae et al. (2016)
Acetaminophen (Paracetamol)			Concentration of *N*-acetyl-*p*-benzoquinone imine NAPQI)-GSH eightfold lower in female liver microsomes than in human liver microsomes	In vitro; ♀ liver microsomes; 2 h; 28.5 °C; 1 mM; UHPLC/MS/MS; UPLC/QTOF/MS/MS	Chng et al. (2012)
Amiodarone			6 mpf Metabolite concentrations in liver microsomes, wild-type ZF *vs*. humanized transgenic zebrafish line: hydroxy-amiodarone, mono-*N*-desmethyl amiodarone, hydroxy-mono-*N*-desmethyl-amiodarone (phase I TPs)	In vivo; 24 h; LC/MS	Poon et al. (2017b)
Benzocaine	~ 27, ~ 51, ~ 75, ~ 99 hpf Benzocaine metabolized to 4-aminobenzoic acid (phase I TP) and 4-acetamidobenzoic acid (phase II TP); 4-aminobenzoic acid likely metabolized into further TPs			In vivo; ≥ 4 hpf, sampling after 24, 48, 72, 96 h; 26 ± 1 °C; 10 to 250 µg/L; HPLC/MS/MS	Brox et al. (2016b)
Benzophenone-2 (BP2)	96 hpf Five phase II metabolites found in larvae extracts: BP2-monoglucuronides, BP2-monosulfate; BP2-disulfate, BP2 double-conjugate: glucuronide and sulfate Same metabolites found as in adult ZF extracts except BP2-diglucuronide Glucuronidation is the major pathway in ZF larvae		Six phase II metabolites found in adult zebrafish extracts: BP2-diglucuronide, two distinct BP2-monoglucuronides, BP2-monosulfate, BP2-disulfate, BP2-double conjugate: glucuronide and sulfate Biotransformation of PB2 was stronger in adults Sulfation major pathway in adult ZF; several conjugates released into water	In vivo; adult male ZF; 96 h; 28 ± 2 °C; 1 µM; Radio-HPLC	Le Fol et al. (2017b)
Benzotriazoles (4-Methyl-1-H-benzotriazole, 1-H-benzotriazole, 5-methyl-1-H-benzotriazole)	96 hpf Overall identification of 26 TPs (22 reported for the first time); hydroxylated, sulfate conjugated and glucuronic acid conjugated TPs			In vivo, 10 µg/ml, from 96 hpf: 30 s, 2 h, 4 h, 8 h, 24 h, 28 °C, UPLC-Q-TOF-HRMS/MS and HILIC	Damalas et al., 2018
Berberine			Adult ZF extracts: TPs by demethylation (phase I), sulfation and glucuronidation (phase II). Metabolism similar to humans	In vivo; mixed sex; 24 h; 27 ± 1 °C; 20 µM; UHPLC/MS	Li et al. (2015)
Bisphenol S (BPS)	96 hpf No phase I TPs found. Phase II TPs: BPS-mono-glucuronide, BPS-mono-sulfate (major TP)		No phase I TPs found. Phase II TPs: BPS-mono-glucuronide, BPS-mono-sulfate (major TP)	In vivo; adult male ZF; 96 h; 28 ± 2 °C; 1 µM; Radio-HPLC	Le Fol et al. (2017b)
Bupropion		7 dpf Hydroxybupropion (phase I TP) found in ZF homogenate and water		In vivo; 3 h; 26—28 °C; 30 µM; HPLC/MS/MS	Alderton et al. (2010)
Calycosin	54, 60, 66, 72 hpf 7 out of 10 metabolites (phase I and phase II) detected continuously, reactions included hydroxylation, glucuronidation, sulfation, glycosylation			In vivo; 24 h from 72 hpf; 28.5 °C; 30 µM; HPLC/MS/MS	Hu et al. (2012)
Caffeine	50, 120 hpf 1,7-dimethylxanthine (phase I TP) Higher concentration in 120 h old ZF embryos (0.0355 ± 0.0069 ng per whole embryo) than in 50 hpf ZF embryos (0.0161 ± 0.0025 ng per whole embryo)			In vivo*,* 24 h, 28 ± 1 °C 10 mg/L (0.01% DMSO) LC–MS	Nawaji et al. (2020)
Cisapride	3 dpf No metabolite found	7 dpf Phase II: cisapride *N*-sulfate Major mammalian phase I and II TPs not found		In vivo; 1/3 h; 26—28 °C; 50/500 µM; HPLC/MS/MS	Alderton et al. (2010)
Clofibric acid	7, 10, 28, 52, 76, 100 hpf Phase I and II TPs formed: sulfated TP from 7 to10 hpf. Majority of 18 TPs after 28 hpf. Sulfate and glucuronide conjugates ≥ 52 hpf. Further phase II conjugates: carnitine, taurine conjugates and aminomethane sulfonate			In vivo; from 4 hpf, sampling after 24, 48, 72 and 96 h; 26 ± 1 °C; 50 mg/L; HPLC/QTOF/MS	Brox et al. (2016a)
Coptisine			Adult ZF extracts: phase I TPs by demethylation and reduction, no phase II TPs	In vivo; mixed sex; 24 h; 27 ± 1 °C; 2 µM; UHPLC/MS	Li et al. (2015)
Dextromethorphan	5, 24, 48, 72, 96, 120 hpf Dextrorphan in microsomes until 48 hpf under the limit of detection. Significant higher dextrorphan levels in microsomes at 96 hpf than at 120 hpf 3-Methoxymorphinan below the lower limit of detection in all stages except 96 hpf		Adult microsomes from both sexes: Dextromethorphan metabolized into 3-methoxymorphinan and dextrorphan Same metabolites as in humans (at different ratios) No sex-related differences	In vitro; adult liver microsomes from 10 adult ZF (mixed sex) and whole-body microsomes of ~ 1500 embryos; 2 h; 28.5 °C; 10 µM; UPLC/MS/MS	Saad et al. (2017)
Diclofenac	5—72, 96 hpf No metabolites detected In two batches, levels of 4’-hydroxydiclofenac and 5’-hydroxydiclofenac close to limit of detection (both metabolites ~ 10 × lower than in adult ZF liver microsomes)		Hydroxy diclofenac (no difference between female and male ZF liver microsomes)	In vitro; liver microsomes from 10 adult ZF (mixed sex) and whole-body microsomes of ~ 1500 embryos; 2 h; 28.5 °C; 12 µM; UPLC/MS/MS	Saad et al. (2017)
Diclofenac	24, 48, 72, 96 and 120 hpf Concentration of 4’hydroxydiclofenac reached maximum at 72 hpf (8.90 ± 0.21 ng/embryo) Concentration of 5’-hydroxydiclofenac reached maximum at 96 hpf (2.80 ± 0.31 ng/embryo)			In vivo*,* 24 h, 28 ± 1 °C 10 mg/L (0.01% DMSO) LC–MS/MS	Nawaji et al. (2020)
Diclofenac		7 dpf 0.6% of the parent compound as hydroxy diclofenac in larval homogenates (mean: 2.3 µM)		In vivo; 3 h; 26—28 °C; 30 µM; LC/MS/MS	Alderton et al. (2010)
Febantel		144 hpf Febantel and corresponding phase I metabolites fenbendazole and oxfendazole in exposure medium		In vivo; 7 d; 25 ± 0.4 °C; 0.02—2.0 mg/L (4.8—4480 nM); SPE-LC–MS/MS	Carlsson et al. (2013)
Fluoxetine	72, 82, 94, 96, 120 hpf Norfluoxetine dominant metabolite. 11 metabolites: aromatic hydroxylation, *N*-hydroxylation., *N*-acylation, *N*-formylation, *N*-methylation, *N*-propionylation, *N*-fumarylation, conjugation with L-valine			In vivo; 48—120 hpf; 26 ± 1 °C; 10, 50, 5000 µg/L; Q-TOF LC/MS	Zindler et al. (2020)
Ibuprofen	78 hpf: No metabolites detected after 6-h exposure 96 hpf: hydroxy-ibuprofen, traces of a second putative hydroxy-ibuprofen metabolite co-eluting with the parent compound, minor unknown metabolite detected in larval extracts only			In vivo; 24 h from 72 hpf; 28 ± 1 °C; 100 µg/L; LC/MS/MS	Jones et al. (2012)
Jatrorrhizine			Phase I and II metabolites including demethylation, methylation, hydroxylation, sulfation and glucuronidation	In vivo (mixed sex); 24 h; 27 ± 1 °C; 20 µM; LC/UHPLC-orbitrap MS	Li et al. (2015)
Lauric acid		7 dpf Significant metabolism of ^14^C- lauric acid to a more polar metabolite (not further identified)		In vivo; 3 h; 28.5 ± 0.1 °C; 100 µM; HPLC, LC/MS/MS	Alderton et al. (2010)
Midazolam			Humanized transgenic ZF line more active than wild-type ZF; phase I and II metabolites detected ZF liver samples: 1’-hydroxy-midazolam, 4’-hydroxy-midazolam, *N*- and *O*-glucuronides of midazolam and hydroxy-midazolam	In vivo; 6 mo; ZF liver; 6 h; 10 µM; LC/MS/MS	Poon et al. (2017b)
Nefazodone			Humanized transgenic ZF line more active than wild-type ZF: Hydroxy nefazodone as major metabolite	In vivo; 6 mo; ZF liver; 6 h; 10 µM; LC/MS/MS	Poon et al. (2017b)
Palmatine			Phase I and II reactions included demethylation, hydroxylation, glucuronidation and sulfation	In vivo (mixed sex); 24 h; 27 ± 1 °C; 20 µM; LC/UHPLC-orbitrap MS	Li et al. (2015)
Phenacetin	~ 28, ~ 52, ~ 76, ~ 100 hpf 3 metabolites: paracetamol (phase I TP) with maximum at ~ 28 hpf, paracetamol sulfate and glucuronide (phase II TPs) increased with time			In vivo; ≥ 4 hpf, sampling after 24, 48, 72 and 96 h; 26 ± 1 °C; 10—250 mg/L; HPLC/MS/MS	Brox et al. (2016b)
Phenacetin		7 dpf Hydroxylated tacrine (phase I TP)		In vivo; 3 h; 28.5 ± 0.1 °C; 100 µM; HPLC, LC/MS/MS	Alderton et al. (2010)
Tacrine		7 dpf Hydroxylated tacrine (phase I TP)		In vivo; 3 h; 28.5 ± 0.1 °C; 30 µM; HPLC, LC/MS/MS	Alderton et al. (2010)
Testosterone	5, 24, 48, 72, 96, 120 hpf No testosterone consumption detected		Detection of 6 minor metabolites with several isomers (none dominating). Female ZF 3 × more active than male ZF	In vitro; liver microsomes from 10 ♀ or 10 ♂ ZF and whole-body microsomes of ~ 1500 embryos; 120 min; 28.5 °C; 40 µM; LC-amMS	Saad et al. (2017)
Testosterone	50, 120 hpf No detection of 6β-hydroxytestosterone			In vivo*,* 24 h, 28 ± 1 °C 10 mg/L (0.01% DMSO) LC–MS	Nawaji et al. (2020)
Testosterone	96 hpf Low concentrations or absence of metabolites		Hydroxytestosterone (main human metabolite) not detected. Several isomeric metabolites of C_19_H_39_O, C_19_H_28_, C_19_H_30_O_2,_, C_19_H_32_O_3_, C_25_H_28_O_3_, C_18_H_40_O_9_, and C_26_H_42_O_9_ detected with differences in ♂ and ♀ microsomes	In vitro; liver microsomes from 10 ♀ or 10 ♂ ZF and whole-body microsomes of 96 hpf embryos; 2 h; 28.5 °C; 40 µM; UPLC-amMS	Saad et al. (2018)
Testosterone	5 dpf Two putatively hydroxylated testosterone metabolites in homogenates and media. Major metabolite unique (not found in adult ♀liver microsomes; not further identified). Second-most metabolite: 6β-hydroxytestosterone (phase I TP) Testosterone glucuronide (phase II) detected in embryo homogenates and media samples		7 hydroxylated (phase I) metabolites including 2α-, 6β- and 16β-hydroxytestosterone, 3 putative metabolites (not further identified). Major metabolite: 6β-hydroxytestosterone Third-most metabolite in ♀ ZF liver microsomes not observed in human liver microsomes	In vitro*/*in vivo; 5 dpf ZF and ♀ liver microsomes, 0, 1, 2 h/3 h; 28.5 °C;100 µM/10 µM; UHPLC/MS/MS and UPLC/QTOF/MS/MS	Chng et al. (2012)
Testosterone		7 dpf Hydroxylated testosterone (phase I TP) and testosterone-glucuronide (phase II TP) in larval homogenates		In vivo; 3 h; 28.5 ± 0.1 °C; 10 µM; HPLC, LC/MS/MS	Alderton et al. (2010)
Triphenyl phosphate			6 Metabolites including main metabolite d_10_-diphenyl phosphate and 5 phase I and II TPs: mono-hydroxylated diphenyl phosphate, mono- and dihydroxylated TPHP and their glucuronides after hydroxylation Failure to detect, e.g., sulfate conjugates after hydroxylation, methoxylated TPs after hydroxylation and hydroxylated TP after glucuronidation Highest concentrations in liver and intestine (brain and muscle: below detection limit)	In vivo; adult ZF; metabolites in water and tissue samples; 3, 7, 11, 14, 16 and 19 d¸ 24 ± 1 °C; 20, 100 µg/L; LC/QTOF	Wang et al. (2016)
Verapamil	3 dpf 10 TPs detected including hydroxylation, *O*-dealkylation and *N*-dealkylation (phase I) and glucuronide after oxidation (phase II)	7 dpf 10 Verapamil-related TPs were detected in both 3 dpf and 7 dpf larvae. Underlying reactions included phase I related reaction (e.g., hydroxylation, O-dealkylation, N-dealkylation) and phase II related reactions such as glucuronide conjugation after oxidation		In vivo; 3 h/1 h; 28.5 ± 0.1 °C; 50 µM; LC/MS/MS	Alderton et al. (2010)

*HILIC* hydrophilic interaction liquid chromatography, *HPLC/MS/MS* high performance liquid chromatography- tandem mass spectrometry, *LC-HRMS* liquid chromatography—high resolution mass spectrometry, *LC/MS* liquid chromatography–mass spectrometry*, mo* month, *Q-TOF LC/MS* Quadrupole time-of-flight liquid chromatography–mass spectrometry, *SPE-LC–MS/MS* solid phase extraction coupled with liquid-chromatography tandem mass spectrometry, *TP* transformation product, *UHPLC-amMS* ultra-high performance liquid chromatography – accurate mass mass spectrometry, *UHPLC-orbitrap MS* ultra high-performance liquid chromatography coupled to Orbitrap mass spectrometry, *UHPLC/MS/MS* ultra high performance liquid chromatography—tandem mass spectrometer, *UPLC/QTOF/MS* ultra-high-performance liquid chromatography-quadrupole time-of-flight mass spectrometry,

Numerous studies have indicated that different developmental stages of zebrafish may vary in their biotransformation capacities, either in respect to rates of metabolite formation (i.e. quantitatively) or in the biotransformation pathways they use (i.e. qualitatively; e.g., Brox et al. 2016; Chng et al. 2012; Saad et al. 2017, 2018). In fact, there is growing evidence that the metabolic competence of zebrafish progressively develops with more phase I and phase II metabolites being detected, and less abundant metabolites being enriched as embryonic development progresses. For instance, Brox et al. (2016a) analyzed the metabolite profile of clofibric acid in extracts of zebrafish embryos. They could show that sulfate-containing (phase II) metabolites are formed from very early on (i.e. 7 hpf), while others such as glucuronide conjugates only reached detectable levels at 52 hpf (Brox et al. 2016a).

Whether juvenile zebrafish dispose a fully developed xenobiotic biotransformation capacity is generally not a subject of debate. To date, however, only little information is available on metabolite formation in juvenile zebrafish (Table 6). The only comprehensive study that focused on metabolite formation in juvenile zebrafish was performed by Alderton et al., (2010). In their study, juvenile zebrafish at 168 hpf could be documented to perform phase I (e.g., oxidation, *N*-dealkylation and *O*-dealkylation) and phase II reactions (e.g., glucuronidation and sulfation) similar to humans with a variety of pharmaceutical compounds. However, the metabolites recovered accounted for a very small fraction of the parent compounds administrated (Alderton et al. 2010). Furthermore, this study provided evidence that the metabolic competence of juvenile zebrafish might at least be quantitatively different from that of zebrafish embryos. While 168-h-old zebrafish were documented to metabolize cisapride to the phase II metabolite cisapride-sulfate, no such metabolite formation was observed in zebrafish embryos at 72 hpf (Alderton et al. 2010).

Comparative studies between zebrafish embryos and adult zebrafish, especially those based on microsomal preparations, are beset with problems regarding the comparability of the results. In particular, when studies use microsomes prepared from whole-body homogenates of zebrafish, as has been commonly done when assessing biotransformation activities in embryonic stages of zebrafish, there is a risk of underestimating the level of biotransformation activity obtained in liver microsomes or in vivo (Verbueken et al. 2018). The fact that zebrafish embryos and adult zebrafish may qualitatively differ in the metabolic pathways they use has been indicated by (Chng et al. 2012). In this in vitro study, differences were documented with respect to phase I biotransformation of testosterone. While in liver microsomes of adult zebrafish testosterone was metabolized to seven hydroxylated metabolites, only two hydroxylated metabolites were detected in whole-body microsomes of 5-d-old zebrafish embryos, whose main metabolite was unique (i.e. not found in adult zebrafish liver microsomes nor in human liver microsomes (Chng et al. 2012)). However, as indicated by studies of Saad et al. (2017, 2018), the metabolism of testosterone varies not only with age, but also with sex.

Quantitatively different, but qualitatively similar biotransformation activities in zebrafish embryos and adult zebrafish have been documented in an in vivo study by Le Fol et al. (2017). They documented that the biotransformation of benzophenone-2 was more extensive in adult zebrafish, if compared to embryos. However, expect for a single phase II metabolite, i.e. benzophenone-2-diglucuronide, which was only detected in adult zebrafish, five identical metabolites were produced in both developmental stages (Le Fol et al. 2017).

## Conclusions and recommendations

Embryonic, juvenile and adult stages of zebrafish have been studied to a very different extent with respect to their biotransformation and bioactivation capacities. Especially juvenile zebrafish have been neglected so far. As a consequence, our knowledge about the development of the xenobiotic biotransformation capacity in zebrafish can be called—at best—fragmentary.

At the mRNA level, there is ample evidence that the vast majority of zebrafish CYP1, CYP2, CYP3 and CYP4 genes are constitutively expressed from earliest embryonic stages of development. At a first glance, this might be interpreted as an indication of a fundamental existence of an early competence for xenobiotic biotransformation. Since, however, extrapolation of gene expression levels to biochemical functionality is technically not possible to date, it is questionable whether this interpretation holds. One of the fundamental questions remaining to clarified is whether or not the developmental- and sex-related differences in CYP1 to 4 mRNA expression levels will lead to age- and sex-related differences in xenobiotic biotransformation capacities and, eventually, to differences in outcomes of toxicological studies.

**Fig. 5:**
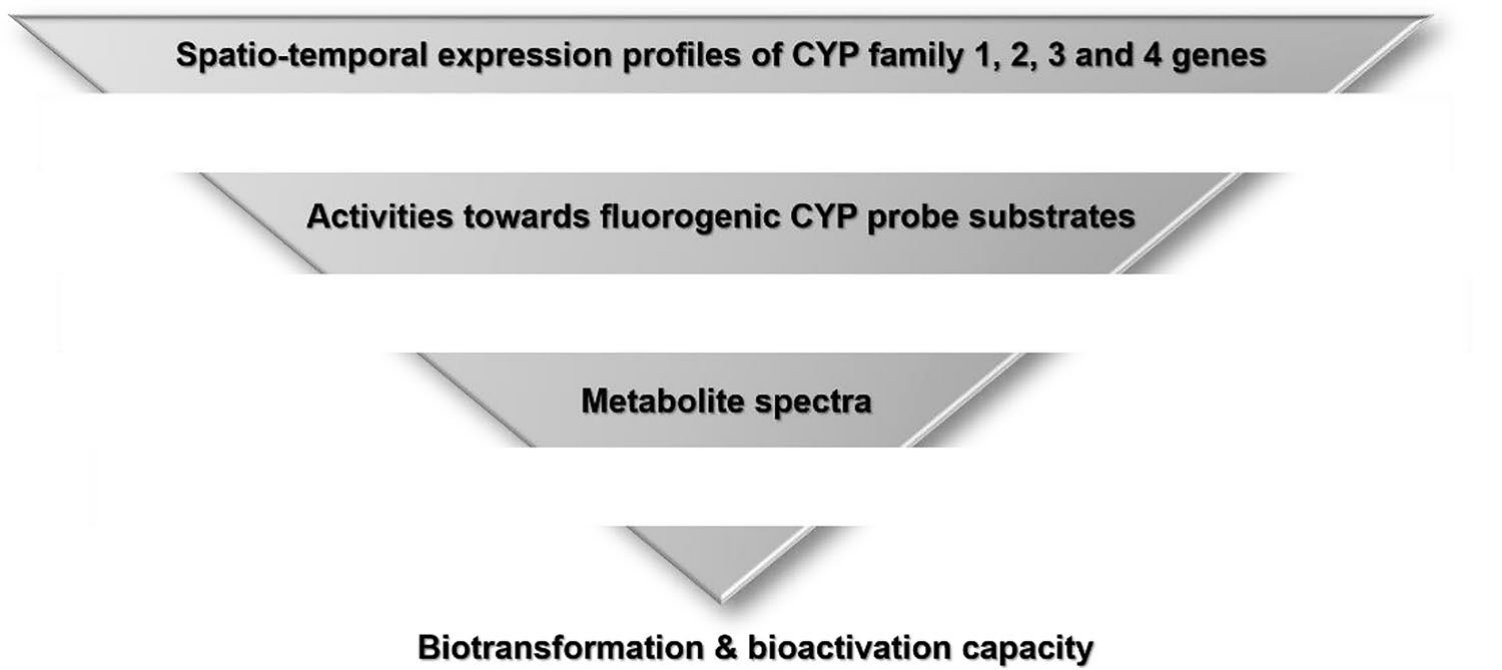
Availability of data on the cytochrome P450 system and phase I biotransformation in zebrafish (*Danio rerio*)

Overall, studies on CYP gene expression patterns are not as conclusive as studies providing indirect or direct evidence of functional biotransformation activities. However, with respect to CYP-dependent activities, our knowledge is even far more fragmentary as it is for CYP expression patterns in zebrafish (Fig. 5). This is due to the fact that only very few studies allow quantitative comparisons with regard to CYP activities at different developmental stages of zebrafish. Moreover, the diversity of methodological approaches between studies does not allow an adequate comparison of results.

Evidence on the functionality of the CYP-system in zebrafish have come from studies monitoring formation of fluorescent and luminescent metabolites or assessing effects of mammalian pro-toxicants and pro-teratogens known to be activated by CYP enzymes in mammals. Again, there is evidence that zebrafish embryos from earliest stages of development do have functional biotransformation pathways. In many cases, the data available suggest that the biotransformation pathways of zebrafish embryos are at least qualitatively similar to those of juvenile and adult zebrafish as well as those of humans. Only for rare exceptions, such as in case of allyl alcohol, a lack of biotransformation could be found, which was due to a lack of the enzyme required for bioactivation. There is a need for systematic screening from which developmental stages CYP-dependent biotransformation capacities are sufficiently developed to biotransform and bioactivate xenobiotic compounds to a biologically meaningful extent. However, even when formation of a metabolite, bioactivated or not, is documented, the question remains whether the concentration of a metabolite—or the concentration of the parent compound—is sufficiently high to exert a toxicological effect in the respective developmental stage or sex of (adult) zebrafish.

There is a growing number of studies that use chemical analyses to obtain quantitative and/or qualitative information on biotransformation pathways and activities. Such studies, especially those examining full metabolite spectra, are a major challenge in terms of expertise, resources, and funding, but can assist in obtaining much better knowledge and more in-depth understanding of, e.g., the substrates accepted by zebrafish CYP isoforms and factors (e.g., age and sex) that may influence biotransformation activities and, eventually, the outcome of toxicological studies.

## Abbreviations

BFC: 7-Benzyloxy-4-(trifluorormethyl) coumarin
BOMR: Benzyloxymethylresorufin-*O*-deethylase
BR: 7-Benzyloxyresorufin
BROD: 7-Benzyloxyresorufin-*O*-debenzylase
CYP: Cytochrome P450-dependent monooxygenases
dpf: Days post-fertilization
EC: 7-Ethoxycoumarin
ECOD: 7-Ethoxycoumarin-*O*-deethylase
eGFP: Enhanced green fluorescent protein
ER: 7-Ethoxyresorufin
EROD: 7-Ethoxyresorufin-*O*-deethylase
HPLC/MS/MS: High-performance liquid chromatography–tandem mass spectrometry
hpf: Hours post-fertilization
IHC: Immunohistochemistry
ISH: In situ hybridization
LC-HRMS: Liquid chromatography–high-resolution mass spectrometry
LC/MS: Liquid chromatography–mass spectrometry
luciferin-BE: Luciferin-6’-benzylether
luciferin-IPA: Luciferin-isopropyl acetal
luciferin-PFBE: Luciferin-6’-pentafluorobenzyl
MC: 7-Methoxycoumarin
MCOD: 7-Methoxycoumarin-*O*-demethylase
Mo: Month
MP: Microsomal protein
mpf: Months post-fertilization
n.s.: Non specified
OOMR: *N*-octyloxymethylresorufin
PR: 7-Pentoxyresorufin
PROD: 7-Pentoxyresorufin-*O*-depentylase
qPCR: Quantitative real-time reverse transcription polymerase chain reaction
Q-TOF-LC/MS: Quadrupole time-of-flight liquid chromatography – mass spectrometry
RS: Resorufin
SPE: Solid phase extraction
TL: Transgenic lines
TP: Transformation product
UHPLC-amMS: Ultra-high-performance liquid chromatography–accurate mass spectrometry
WISH: Whole mount in situ hybridization
zf: Zebrafish

## References

[CR1] Abrahamson A, Andersson C, Jönsson ME, Fogelberg O, Orberg J, Brunström B, Brandt I (2007). Gill EROD in monitoring of CYP1A inducers in fish − a study in rainbow trout (*Oncorhynchus mykiss*) caged in Stockholm and Uppsala waters. Aquat Toxicol.

[CR2] Alderton W, Berghmans S, Butler P, Chassaing H, Fleming A, Golder Z, Richards F, Gardner I (2010). Accumulation and metabolism of drugs and CYP probe substrates in zebrafish larvae. Xenobiotica.

[CR3] Andersson T, Goksøyr A (1994). Distribution and induction of cytochrome P450 1A1 in the rainbow trout brain. Fish Phyiol Biochem.

[CR4] Auerbach SS, Mahler J, Travlos GS, Richard D (2008). A comparative 90 day toxicity study of allyl acetate, allyl alcohol and acrolein. Toxicology.

[CR5] Bakkers J (2011). Zebrafish as a model to study cardiac development and human cardiac disease. Cardiovasc Res.

[CR6] Bambino K, Chu J (2017). Zebrafish in toxicology and environmental health. Curr Top Dev Biol.

[CR7] Barros TP, Alderton WK, Reynolds HM, Roach AG, Berghmans S (2008). Zebrafish: an emerging technology for *in vivo* pharmacological assessment to identify potential safety liabilities in early drug discovery. Br J Pharmacol Chemother.

[CR8] Boehler S, Lörracher AK, Schubert J, Braunbeck T (2018). Comparative live-imaging of *in vivo* EROD (ethoxyresorufin-*O*-deethylase) induction in zebrafish (*Danio rerio*) and fathead minnow (*Pimephales promelas*) embryos after exposure to PAHs and river sediment extracts. Sci Total Environ.

[CR9] Braunbeck T, Kais B, Lammer E, Otte J, Schneider K, Stengel D, Strecker R (2015). The fish embryo test (FET): origin, applications, and future. Environ Sci Pollut Res.

[CR10] Bräunig J, Schiwy S, Broedel O, Müller Y, Frohme M, Hollert H, Keiter SH (2015). Time-dependent expression and activity of cytochrome P450 1s in early life-stages of the zebrafish (*Danio rerio*). Environ Sci Pollut Res.

[CR11] Brox S, Seiwert B, Haase N, Küster E, Reemtsma T (2016). Metabolism of clofibric acid in zebrafish embryos (*Danio rerio*) as determined by liquid chromatography-high resolution-mass spectrometry. Comp Biochem Phys C Toxicol Pharmacol.

[CR12] Brox S, Seiwert B, Küster E, Reemtsma T (2016). Toxicokinetics of polar chemicals in zebrafish embryo (*Danio rerio*): Influeice of physicochemical properties and of biological processes. Environ Sci Technol.

[CR13] Bundesgesetzblatt Bundesministerium für Umwelt, Naturschutz und Reaktorsicherheit (2005) Bekanntmachung der neufassung des abwasserabgabegesetzes. Teil I, Nr. 5, Bonn, 25. http://extwprlegs1.fao.org/docs/pdf/ger35872b.pd

[CR14] Burkina V, Sakalli S, Zamaratskaia ZV (2018). CYP1A1 activity in rainbow trout is inhibited by the environmental pollutant *p*-cresol. Environ Toxicol Pharmacol.

[CR15] Busquet F, Nagel R, von Landenberg F, Mueller SO, Huebler N, Broschard TH (2008). Development of a new screening assay to identify proteratogenic substances using zebrafish (*Danio rerio*) embryo combined with an exogenous mammalian metabolic activation system (mDarT). Toxicol Sci.

[CR16] Busquet F, Halder BT, Gourmelon LA, Kleensang A, Belanger S, Carr GJ, Walter-Rohde S (2013). OECD guidelines for the testing of chemicals 236 – fish embryo acute toxicity (FET) test. The OECD Observer. Organ Econ Co-Oper Dev.

[CR17] Cali JJ, Ma D, Sobol M, Simpson DJ, Frackman S, Good TD, Daily WJ, Liu D (2006). Luminogenic cytochrome P450 assays. Expert Opin Drug Metabol Toxicol.

[CR18] Cali JJ, Sobol M, Ma D, Uyeda HAT, Meisenheimer P (2009) CYP3A4 P450-Glo^®^ Assays with luciferin-IPA: the most sensitive and selective bioluminescent CYP3A4 assay. Promega Corporation Web site. https://www.promega.de/en/resources/pubhub/cellnotes/cyp3a4-p450-glo-assays-with-luciferin-ipa-the-most-sensitive-and-selective-bioluminescent-cyp3a4/. (Accessed 12 Dec 2020).

[CR19] Carlsson G, Patring J, Kreuger J, Norrgren L, Oskarsson A (2013). Toxicity of 15 veterinary pharmaceuticals in zebrafish (*Danio rerio*) embryos. Aquat Toxicol.

[CR20] Chang CT, Chung HY, Su HT, Tseng HP, Tzou WS, Hu CH (2013). Regulation of zebrafish CYP3A65 transcription by AHR2. Toxicol Appl Pharmcol.

[CR21] Chen CH (2020) Functionalization reactions catalyzed by activation enzymes. In: Chen C-H (ed) Xenobiotic metabolic enzymes:bioactivation and antioxidant defense. Springer, pp 59–70

[CR22] Chng HT (2013) Evaluation of alternative *in vivo* and *in vitro* models for drug metabolism testing in drug discovery. National University of Singapore

[CR23] Chng HT, Ho HK, Yap CW, Lam SH, Chan ECY (2012). An investigation of the bioactivation potential and metabolism profile of zebrafish versus human. J Biomol Screen.

[CR24] Corley-Smith GE, Su HT, Wang-Buhler JL, Tseng HP, Hu CH, Hoang T, Chung WG, Buhler DR (2006). CYP3C1, the first member of a new cytochrome P450 subfamily found in zebrafish *(Danio reri*o) Biochem. Biophys Res Commun.

[CR25] Creusot N, Brion F, Piccini B, Budzinski H, Porcher JM, Ait-Aissa S (2015). BFCOD activity in fish cell lines and zebrafish embryos and its modulation by chemical ligands of human aryl hydrocarbon and nuclear receptors. Environ Sci Pollut Res.

[CR26] Damalas DE, Bletsou AA, Agalou A, Beis D, Thomaidis NS (2018). Assessment of the acute toxicity, uptake and biotransformation potential of benzotriazoles in zebrafish (*Danio rerio)* larvae combining HILIC- with RPLC-HRMS for high-throughput identification. Enciron Sci Technol.

[CR27] De Almeida L, Froneman W, Pletschke B (2011). Optimization of a cytochrome-P450-monooxygenase-1A-mediated EROD assay in the cape hake species *Merluccius capensis* and *Merluccius paradoxus* (pisces). Enzyme Res.

[CR28] Dohnal V, Wu QH, Kuca K (2014). Metabolism of aflatoxins: key enzymes and interindividual as well as interspecies differences. Arch Toxicol.

[CR29] Doshi U, Li AP (2011). Luciferin IPA-based higher throughput human hepatocyte screening assays for CYP3A4 inhibition and induction. J Biomol Screen.

[CR30] ECHA (2017) Expert workshop on the potential regulatory application of the fish embryo acute toxicity (FET). Test under REACH, CLP and the BPR

[CR31] EU (2010) Directive 2010/63/EU of the European parliament and of the council of 22 September 2010 on the protection of animals used for scientific purposes L276. Official Journal of the European Union, pp 33–79.

[CR32] Fetter E, Smetanova S, Baldauf LA, Altenburger R, Schüttler A, Scholz S (2015). Identification and characterization of androgen-responsive genes in zebrafish embryos. Environ Sci Technol.

[CR33] Funari E, Zoppini A, Verdina A, Angelis GD, Vittozzi L (1987). Xenobiotic-metabolizing enzyme systems in test fish. I. Comparative-studies of liver microsomal monooxygenases. Ecotox Environ Saf.

[CR34] Glass AS, Dahm R (2004). The zebrafish as a model organism for eye development. Ophthalmic Res.

[CR35] Glisic B, Hrubik J, Fa S, Dopudj N, Kovacevic R, Andric N (2016). Transcriptional profiles of glutathione-S-transferase isoforms, Cyp, and AOE genes in atrazine-exposed zebrafish embryos. Environ Toxicol.

[CR36] Goldstone JV, Jönsson ME, Behrendt L, Woodin BR, Jenny MJ, Nelson DR, Stegeman JJ (2009). Cytochrome P450 1D1: a novel CYP1A-related gene that is not transcriptionally activated by PCB126 or TCDD. Arh Biochem Biophys.

[CR37] Goldstone JV, McArthur AG, Kubota A, Zanette J, Parente T, Jönsson ME, Nelson DR, Stegeman JJ (2010). Identification and developmental expression of the full complement of cytochrome P450 genes in zebrafish. BMC Genom.

[CR38] González-Doncel M, San Segundo L, Sastre S, Tarazona JV, Torija CF (2011). Dynamics of BNF-induced *in vivo* ethoxyresorufin-*O*-deethylase (EROD) activity during embryonic development of medaka (*Oryzias latipes*). Aquat Toxicol.

[CR39] Guengerich FP (2001). Common and uncommon cytochrome P450 reactions related to metabolism and chemical toxicity. Chem Res Toxicol.

[CR40] Guengerich FP (2003). Cytochromes P450, drugs, and diseases. Mol Interv.

[CR41] Guengerich FP (2017). Intersection of the roles of cytochrome P450 enzymes with xenobiotic and endogenous substrates: Relevance to toxicity and drug interactions. Chem Res Toxicol.

[CR42] Hegelund T, Celander MC (2003). Hepatic versus extrahepatic expression of CYP3A30 and CYP3A56 in adult killifish (*Fundulus heteroclitus*). Aquat Toxicol.

[CR43] Hill AJ, Teraoka H, Heideman W, Peterson RE (2005). Zebrafish as a model vertebrate for investigating chemical toxicity. Toxicol Sci.

[CR44] Hill DS, Wlodarczyk BJ, Palacios AM, Finnell RH (2010). Teratogenic effects of antiepileptic drugs. Expert Rev Neurother.

[CR45] Hu G, Siu SO, Li S, Chu IK, Kwan YW, Chan SW, Leung GPH, Lee YR, SMY, (2012). Metabolism of calycosin, an isoflavone from *Astragali Radix*, in zebrafish larvae. Xenobiotica.

[CR46] Isin EM, Guengerich FP (2006). Complex reactions catalyzed by cytochrome P450 enzymes. Biochim Biophys Acta.

[CR47] ISO (2016). Water quality − Determination of the acute toxicity of waste water to zebrafish eggs (*Danio rerio*). ISO.

[CR48] Jones HS, Panter GH, Hutchinson TH, Chipman JK (2010). Oxidative and conjugative xenobiotic metabolism in zebrafish larvae *in vivo*. Zebrafish.

[CR49] Jones HS, Trollope HT, Hutchinson TH, Panter GH, Chipman JK (2012). Metabolism of ibuprofen in zebrafish larvae. Xenobiotica.

[CR50] Jönsson ME, Jenny MJ, Woodin BR, Hahn ME, Stegeman JJ (2007). Role of AHR2 in the expression of novel cytochrome p450 1 family genes, cell cycle genes, and morphological defects in developing zebrafish exposed to 3,3',4,4',5-pentachlorobiphenyl or 2,3,7,8-tetrachlorodibenzo-*p*-dioxin. Toxicol Sci.

[CR51] Jönsson ME, Orrego R, Woodin BR, Goldstone JV, Stegeman JJ (2007). Basal and 3,3',4,4',5-pentachlorobiphenyl-induced expression of cytochrome P450 1A, 1B and 1C genes in zebrafish. Toxicol Appl Pharmacol.

[CR52] Jönsson ME, Brunström B, Brandt I (2009). The zebrafish gill model: induction of CYP1A, EROD and PAH adduct formation. Aquat Toxicol.

[CR53] Kane DA, Kimmel CB (1993). The Zebrafish midblastula transition. Development.

[CR54] Kantae V, Krekels EHJ, Ordas A, González O, van Wijk RC, Harms AC, Racz PI, van der Graaf PH, Spaink HP, Hankemeier T (2016). Pharmacokinetic modeling of paracetamol uptake and clearance in zebrafish larvae: expanding the allometric scale in vertebrates with five orders of magnitude. Zebrafish.

[CR55] Kim KH, Park HJ, Kim JH, Kim S, Williams DR, Kim MK, Jung YD, Teraoka H, Park HC, Choy HE, Shin BA, Choi SY (2013). CYP1a reporter zebrafish reveals target tissues for dioxin. Aquat Toxicol.

[CR56] Kithcart A, MacRae CA (2017). Using zebrafish for high-throughput screening of novel cardiovascular drugs. J Am Coll Cardiol Basic Trans Sci.

[CR57] Klüver N, Ortmann J, Paschke H, Renner P, Ritter AP, Scholz S (2014). Transient overexpression of adh8a increases allyl alcohol toxicity in zebrafish embryos. PLoS ONE.

[CR58] Klüver N, König M, Ortmann J, Massei R, Paschke A, Kühne R, Scholz S (2015). Fish embryo toxicity test: identification of compounds with weak toxicity and analysis of behavioral effects to improve prediction of acute toxicity for neurotoxic compounds. Environ Sci Technol.

[CR59] Kubota A, Bainy ACD, Woodin BR, Goldstone JV, Stegeman JJ (2013). The cytochrome P450 2AA gene cluster in zebrafish (*Danio rerio*): expression of CYP2AA1 and CYP2AA2 and response to phenobarbital-type inducers. Toxicol Appl Pharmacol.

[CR60] Kubota A, Kawai YK, Yamashita N, Lee JS, Kondoh D, Zhang S, Nishi Y, Suzuki K, Kitazawa T, Teraoka H (2019). Transcriptional profiling of cytochrome P450 genes in the liver of adult zebrafish, *Danio rerio*. J Toxicol Sci.

[CR61] Le Fol V, Brion F, Hillenweck A, Perdu E, Bruel S, Ait-Aissa S, Cravedi JP, Zalko D (2017). Comparison of the *in vivo* biotransformation of two emerging estrogenic contaminants, BP2 and BPS, in zebrafish embryos and adults. Int j Mol Sci.

[CR62] Li C, Luo L, Awerman J, McGrath P (2011) Whole zebrafish cytochrome P450 assay for assessing drug metabolism and safety. In: McGrath P (ed) Zebrafish Methods for Assessing Drug Safety and Toxicity. John Wiley and Sons Ltd, pp 103–115

[CR63] Li Y, Wang H, Si N, Ren W, Han L, Xin S, Zuo R, Wei X, Yang J, Zhao H, Bian B (2015). Metabolic profiling analysis of berberine, palmatine, jatrorrhizine, coptisine and epiberberine in zebrafish by ultra-high performance liquid chromatography coupled with LTQ Orbitrap mass spectrometer. Xenobiotica.

[CR64] Lillicrap A, Moe SJ, Wolf R, Connors KA, Rawlings JM, Landis WG, Madsen A, Belanger SE (2020). Evaluation of a Bayesian network for strengthening the weight of evidence to predict acute fish toxicity from fish embryo toxicity data. Interg Environ Assess Manag.

[CR65] Lin CH, Chou PH, Chen PJ (2014). Two azole fungicides (carcinogenic triadimefon and non-carcinogenic myclobutanil) exhibit different hepatic cytochrome P450 activities in medaka fish. J Hazard Mater.

[CR66] Loerracher AK, Grethlein M, Braunbeck T (2020). *In vivo* fluorescence-based characterization of cytochrome P450 activity during embryonic development of zebrafish (*Danio rerio*). Ecotox Environ Saf.

[CR67] McGrath P, Li CQ (2008). Zebrafish: a predictive model for assessing drug-induced toxicity. Drug Discov.

[CR68] Moe SJ, Madsen AL, Connors KA, Rawlings JM, Belanger SE, Landis WG, Wolf R, Lillicrap AD (2020). Development of a hybrid Bayesian network model for predicting acute fish toxicity using multiple lines of evidence. Environ Model Softw.

[CR69] Nawaji T, Yamashita N, Umeda H, Zhang S, Mizoguchi N, Seki M, Kitazawa T, Teraoka H (2020). Cytochrome P450 expression and chemical metabolic activity before full liver development in zebrafish. Pharmaceuticals.

[CR70] Nebert DW, Russell DW (2002). Clinical importance of the cytochromes P450. Lancet.

[CR71] Nebert DW, Wikvall K, Miller WL (2013). Human cytochromes P450 in health and disease. Philos Trans R Soc B.

[CR72] Nelson DR (2006). Cytochrome P450 nomenclature, 2004. Methods Mol Biol.

[CR73] Nelson DR, Kamataki T, Waxman DJ, Guengerich FP, Estabrook RW, Feyereisen R, Gonzalez FJ, Coon MJ, Gunsalus IC, Gotoh O (1993). The P450 superfamily − update on new sequences, gene-mapping, accession numbers, early trivial names of enzymes, and nomenclature. DNA Cell Biol.

[CR74] Norberg-King TJ, Embry MR, Belanger SE, Braunbeck T, Butler JD, Dorn PB, Farr B, Guiney PD, Hughes SA, Jeffries M, Journal R, Lèonard M, McMaster M, Oris JT, Ryder K, Segner H, Senac T, Van der Kraak G, Whale G, Wilson P (2018). An international perspective on the tools and concepts for effluent toxicity assessments in the context of animal alternatives: reduction in vertebrate use. Environ Toxicol Chem.

[CR75] OECD (1992) OECD guidelines for the testing of chemicals. Section 2: effects on biotic systems OECD. Test guideline 203: Fish Acute toxicity test. Organization for Economic Cooperation and Development

[CR76] OECD (2019) OECD guidelines for the testing of chemical. Section 2: Effects on biotic systems. Test No. 203: Fish Acute Toxicity Test. Organization for Economic Cooperation and Development

[CR77] Ohno Y, Ormstad K, Ross D, Orrenius S (1985). Mechanism of allyl alcohol toxicity and protective effects of low-molecular-weight thiols studied with isolated rat hepatocytes. Toxicol Appl Pharmacol.

[CR78] Ortiz-Delgado JB, Behrens A, Segner H, Sarasquete C (2008). Tissue-specific induction of EROD activity and CYP1A protein in *Sparus aurata* exposed to B(a)P and TCDD. Ecotox Environ Saf.

[CR79] Otte JC, Schmidt AD, Hollert H, Braunbeck T (2010). Spatio-temporal development of CYP1 activity in early life-stages of zebrafish (*Danio rerio*). Aquat Toxicol.

[CR80] Otte JC, Schultz B, Fruth D, Fabian E, van Ravenzwaay B, Hidding B, Slinas ER (2017). Intrinsic xenobiotic metabolizing enzyme activities in early life stages of zebrafish (*Danio rerio*). Toxicol Sci.

[CR81] Oziolor EM, Carey AN, Matson CW (2017). A non-destructive BFCOD assay for *in vivo* measurement of cytochrome P450 3A (CYP3A) enzyme activity in fish embryos and larvae. Ecotox.

[CR82] Paparella M, Scholz S, Belanger S, Braunbeck T, Bicherel P, Connors K, Faßbender C, Halder M, Lillicrap A, Liska R, Schirmer K, Stoddart G, Thomas P, Waler-Rohde S (2021). Limitations and uncertainties of acute fish toxicity assessments can be reduced using alternative methods. Altex.

[CR83] Parente TEM, De-Oliveira ACAX, Paumgartten FJR (2008). Induced cytochrome P450 1A activity in cichlid fishes from Guandu River and Jacarepaguá Lake, Rio de Janeiro. Brazil Environ Pollut.

[CR84] Parkinson A, Ogilvie BW (2008) Biotransformation of xenobiotics. In: Klaassen CD (ed) Casarett and Doull’s toxicology: the basic science of poisons, 7th edn. McGraw-Hill Professional, pp 161–304

[CR85] Parkinson A, Ogilvie B, Buckley D, Kazmi F, Czerwinski M, Parkinson O (2013) Biotransformation of xenobiotics. In: Klaassen CD (ed) Casarett and Doull’s toxicology the basic science of poisons. McGraw-Hill Professional, pp 185–367

[CR86] Pastrakuljic A, Tang BK, Roberts EA, Kalow W (1997). Distinction of CYP1A1 and CYP1A2 activity by selective inhibition using fluvoxamine and isosafrole. Biochem Pharmacol.

[CR87] Pauka LM, Maceno M, Rossi SC, Silva de Assis HC (2011). Embryotoxicity and biotransformation responses in zebrafish exposed to water-soluble fraction of crude oil. Bull Environ Contam Toxicol.

[CR88] Peng X, Shang G, Wang W, Chen X, Lou Q, Zhai G, Li D, Du Z, Ye Y, Jin X, He J, Zhang Y, Yin Z (2017). Fatty acid oxidation in zebrafish adipose tissue is promoted by 1alpha,25(OH)2D3. Cell Rep.

[CR89] Penner N, Woodward C, Prakash C (2012) Appendix: drug metabolizing enzymes and biotransformation reactions. In: Zhang D, Surapaneni S (eds) ADME-enabling technologies in drug design and development. John Wiley ands Sons Inc

[CR90] Poon KL, Wang XG, Lee SGP, Ng AS, Goh WH, Zhao Z, Al-Haddawi M, Wang H, Mathavan S, Ingham PW, McGinnis C, Carney TJ (2017). Transgenic zebrafish reporter lines as alternative *in vivo* organ toxicity models. Toxicol Sci.

[CR91] Poon KL, Wang X, Ng AS, Goh WH, Mcginnis C, Fowler S, Carney TJ, Wang H, Ingham PW (2017). Humanizing the zebrafish liver shifts drug metabolic profiles and improves pharmacokinetics of CYP3A4 substrates. Arch Toxicol.

[CR92] Qiang M, Lu AYH (2014) Drug-metabolizing enzymesA group of promiscuous catalysts. In: Aizawa H, Gan LL, Prakash C, Zhong D, Lee PW (eds) Handbook of metabolic pathways of xenobiotics, pp 1–22

[CR93] Reinecke M, Segner H (1998). Immunohistochemical localization of cytochrome P4501A in developing turbot, *Scophthalmus maximus*. Mar Environ Res.

[CR94] Rendic S, Guengerich FP (2015). Survey of human oxidoreductases and cytochrome P450 enzymes involved in the metabolism of xenobiotic and natural chemicals. Chem Res Toxicol.

[CR95] Renwick AB, Surry D, Price RJ, Lake BG, Evans DC (2000). Metabolism of 7-benzyloxy-4-trifluoromethyl-coumarin by human hepatic cytochrome P450 isoforms. Xenobiotica.

[CR96] Saad M, Cavanaugh K, Verbueken E, Pype C, Casteleyn C, Van Ginneken C, Van Cruchten S (2016). Xenobiotic metabolism in the zebrafish: a review of the spatiotemporal distribution, modulation and activity of Cytochrome P450 families 1 to 3. J Toxicol Sci.

[CR97] Saad M, Verbueken E, Pype C, Casteleyn C, Van Ginneken C, Maes L, Cos P, Van Cruchten S (2016). In vitro CYP1A activity in the zebrafish: temporal but low metabolite levels during organogenesis and lack of gender differences in the adult stage. Reprod Toxicol.

[CR98] Saad M, Matheeussen A, Bijttebier S, Verbueken E, Pype C, Casteleyn C, Van Ginneken C, Apers S, Maes L, Cos P, Van Cruchten S (2017). In vitro CYP-mediated drug metabolism in the zebrafish (embryo) using human reference compounds. Toxicol Vitro.

[CR99] Saad M, Bijttebier S, Matheeussen A, Verbueken E, Pype C, Casteleyn C, Van Ginneken C, Maes L, Cos P, Van Cruchten S (2018). UPLC/MS MS data of testosterone metabolites in human and zebrafish liver microsomes and whole zebrafish larval microsomes. Data Brief.

[CR100] Scholz S, Sela E, Blaha L, Braunback T, Galay-Burgos M, Garciá-Franco M, Guinea J, Klüver N, Schirmer K, Tanneberger K, Tobor-Kapton M, Witters H, Belander S, Benefenati E, Creton S, Cronin MTD, Eggen RIL, Embry M, Ekman D, Gourmelon A, Halder M, Hardy B, Hartung T, Hubesch B, Jungmann D, Lampi MA, Lee L, Léonard M, Küster E, Lillicrap A, Luckenbach T, Murk AJ, Navas JM, Peijnenburg W, Repetto G, Salinas E, Schüürmann SH, Tollefsen KE, Walter-Rohde S, Whale G, Wheeler JR, Winter MJ (2013). A European perspective on alternatives to animal testing for environmental hazard identification and risk assessment. Regul Toxicol Pharmacol.

[CR101] Scornaienchi ML, Thornton C, Willett KL, Wilson JY (2010). Cytochrome P450-mediated 17β-estradiol metabolism in zebrafish (*Danio rerio*). J Endocrinol.

[CR102] Scornaienchi ML, Thornton C, Willett KL, Wilson JY (2010). Functional differences in the cytochrome P450 1 family enzymes from zebrafish (*Danio rerio*) using heterologously expressed proteins. Arch Biochem Biophys.

[CR103] Shaya L, Dejong C, Wilson JY (2014). Expression patterns of cytochrome P450 3B and 3C genes in model fish species. Comp Biochem Phys c.

[CR104] Smith EM (2009) Cytochrome P450 drug metabolism and protein induction and inhibition in fish liver microsomes. McMaster University

[CR105] Smith BR, Brian WR (1991). The role of metabolism in chemical-induced pulmonary toxicity. Toxicol Pathol.

[CR106] Smith EM, Wilson JY (2010). Assessment of cytochrome P450 fluorometric substrates with rainbow trout and killifish exposed to dexamethasone, pregnenolone-16 alpha-carbonitrile, rifampicin, and β-naphthoflavone. Aquat Toxicol.

[CR107] Sobanska M, Scholz S, Nyman AM, Cesnaitis R, Gutierrez Alonso S, Klüver N, Kühne R, Tyle H, de Knecht J, Dang Z, Lundbergh I, Carlon C, De Coen W (2018). Applicability of the fish embryo acute toxicity (FET) test (OECD 236) in the regulatory context of registration, evaluation, authorisation, and restriction of chemicals (REACH). Environ Toxicol Chem.

[CR108] Stegeman JJ, Smolowitz RM, Hahn ME (1991). Immunohistochemical localization of environmentally induced cytochrome P450IA1 in multiple organs of the marine teleost *Stenotomus chrysops* (Scup). Toxicol Appl Pharmacol.

[CR109] Stegeman JJ, Behrendt L, Woodin BR, Kubota A, Lemaire B, Pompon D, Goldstone JV, Urban P (2015). Functional characterization of zebrafish cytochrome P450 family proteins expressed in yeast. Biochim Biophys. Acta.

[CR110] Stiborová M, Frei E, Schmeiser HH (1992). Comparison of cytochrome P-450- and peroxidase-mediated activations of carcinogenic azo dyes and *N*-nitrosamines. Gen Physiol Biophys.

[CR111] Strähle U, Scholz S, Geisler GP, Hollert H, Rastegar S, Schumacher A, Selderslaghs I, Weiss C, Witters H, Braunbeck T (2012). Zebrafish embryos as an alternative to animal experiments − a commentary on the definition of the onset of protected life stages in animal welfare regulations. Reprod Toxicol.

[CR112] Stresser DM, Turner SD, Blanchard AP, Miller VP, Crespi CL (2002). Cytochrome P450 fluorometric substrates: Identification of isoform-selective probes for rat CYP2D2 and human CYP3A4. Drug Metab Dispos.

[CR113] Suter G (2008) Ecotoxicology. In: Newman MC, Clements WH (eds) Ecotoxicology: a comprehensive treatment. CRC Press Taylor and Francis Group, pp 27–28

[CR114] Taavitsainen P, Honkakoski P, Juvonen R, Pelkonen O, Raunio H (2002) Role of xenobiotic metabolism in drug discovery and development. In: Unbehauen HD (ed) Knowledge for sustainable development volume i chapter physical sciences engineering and technology resources theme control systems, robotics, and automation. Encyclopedia of Life Support Systems (EOLSS) eolss Publisher

[CR115] Taylor AE (2005) Immunohistochemical localization of cytochrome P450s 1A, 2K6, 2K7, 3A65 and 3C1 and expression of P4501A in tumor sensitive and resistant lines of juvenile zebrafish. Oregon State University

[CR116] Testa B (2008) Chapter 32 Biotransformation reactions and their enzymes. In: Wermuth CG (ed) The practice of medicinal chemistry. Academic Press, pp 655–673

[CR117] Tierbach A, Groh KJ, Schöneberger R, Schirmer K, Suter MJF (2020). Biotransformation capacity of zebrafish (Danio rerio) early life stages: functionality of the Mercapturic acid pathway. Toxicol Sci.

[CR118] Tseng HP, Hseu TH, Buhler DR, Wang WD, Hu CH (2005). Constitutive and xenobiotics-induced expression of a novel CYP3A gene from zebrafish larva. Toxicol Appl Pharmacol.

[CR119] Verbueken E, Alsop D, Saad MA, Pype C, Van Peer EM, Casteleyn CR, Van Ginneken CJV, Wilson J, Van Cruchten SJ (2017). In vitro biotransformation of two human CYP3A probe substrates and their inhibition during early zebrafish development. Int J Mol Sci.

[CR120] Verbueken E, Bars C, Ball JS, Periz-Stanacey J, Marei WFA, Tochwin A, Gabrielis IJ, Michiels EDG, Stinckens E, Vergauwen L, Knapen D, Van Ginneken CJ, Van Cruchten SJ (2018). From mRNA expression of drug disposition genes to in vivo assessment of CYP-mediated biotransformation during zebrafish embryonic and larval development. Int J Mol Sci.

[CR121] Wang L, Yao JH, Chen L, Chen J, Xue J, Jia W (2007). Expression and possible functional roles of cytochromes P450 2J1 (zfCyp 2J1) in zebrafish. Biochem Biophys Res Commun.

[CR122] Wang G, Du Z, Chen H, Su Y, Gao S, Mao L (2016). Tissue-specific accumulation, depuration, and transformation of triphenyl phosphate (TPHP) in adult zebrafish (*Danio rerio*). Environ Sci Technol.

[CR123] Wang-Buhler JL, Lee SJ, Chung WG, Stevens JF, Tseng HP, Hseu TH, Hu CH, Westerfield M, Yang YH, Miranda CL, Buhler DR (2005). CYP2K6 from zebrafish (*Danio rerio*): cloning, mapping, developmental/tissue expression, and aflatoxin B1 activation by baculovirus-expressed enzyme. Comp Biochem Phys.

[CR124] Weigt S, Huebler N, Strecker R, Braunbeck T, Broschard T (2011). Zebrafish (*Danio rerio*) embryos as a model for testing proteratogens. Toxicology.

[CR125] Whyte JJ, Jung RE, Schmitt CJ, Tillitt DE (2000). Ethoxyresorufin-*O*-deethylase (EROD) activity in fish as a biomarker of chemical exposure. Crit Rev Toxicol.

[CR126] Wienkers LC, Heath TG (2005). Predicting in vivo drug interactions from in vitro drug discovery data. Nat Rev Drug Discov.

[CR127] Wu H, Gao C, Guo YP, Zhang Y, Zhang J, Ma E (2014). Acute toxicity and sublethal effects of fipronil on detoxification enzymes in juvenile zebrafish (*Danio rerio*). Pestic Biochem Phys.

[CR128] Yamaori S, Araki N, Shionoiri M, Ikehata K, Kamijo S, Ohmori S, Watanabe K (2018). A specific probe substrate for evaluation of CYP4A11 activity in human tissue microsomes and a highly Selective CYP4A11 inhibitor: luciferin-4A and epalrestat. J Pharmacol Exp Ther.

[CR129] Yin HC, Tseng HP, Chung HY, Ko CY, Tzou WS, Buhler DR, Hu CH (2008). Influence of TCDD on zebrafish CYP1B1 transcription during development. Toxicol Sci.

[CR130] Zheng W, Xu H, Lam SH, Luo H, Karuturi RKM, Gong Z (2013). Transcriptomic analyses of sexual dimorphism of the zebrafish liver and the effect of sex hormones. PLoS ONE.

[CR131] Zindler F, Tisler S, Loerracher AK, Zwiener C, Braunbeck T (2020). Norfluoxetine is the only metabolite of fluoxetine in zebrafish (<i>Danio rerio</i>) embryos that accumulates at environmentally relevant exposure scenarios. Environ Sci Technol.

[CR132] Zlabek V, Zamaratskaia G (2012). Comparison of three fluorescent CYP3A substrates in two vertebrate models: pig and Atlantic salmon. Animal.

